# Functional Analysis of Genes *GlaDFR1* and *GlaDFR2* Encoding Dihydroflavonol 4-Reductase (DFR) in *Gentiana lutea* L. Var. *Aurantiaca* (M. Laínz) M. Laínz

**DOI:** 10.1155/2022/1382604

**Published:** 2022-01-10

**Authors:** Tingting Yu, Guojun Han, Zhihui Luan, Changfu Zhu, Jinghua Zhao, Yanmin Sheng

**Affiliations:** School of Life Sciences, Changchun Normal University, Changchun 130031, China

## Abstract

Anthocyanins are important pigments for flower color, determining the ornamental and economic values of horticultural plants. As a key enzyme in the biosynthesis of anthocyanidins, dihydroflavonol 4-reductase (DFR) catalyzes the reduction of dihydroflavonols to generate the precursors for anthocyanidins (i.e., leucoanthocyanidins) and anthocyanins. To investigate the functions of DFRs in plants, we cloned the *GlaDFR1* and *GlaDFR2* genes from the petals of *Gentiana lutea* var. *aurantiaca* and transformed both genes into *Nicotiana tabacum* by *Agrobacterium*-mediated leaf disc method. We further investigated the molecular and phenotypic characteristics of T1 generation transgenic tobacco plants selected based on the hygromycin resistance and verified by both PCR and semiquantitative real-time PCR analyses. The phenotypic segregation was observed in the flower color of the transgenic tobacco plants, showing petals darker than those in the wild-type (WT) plants. Results of high-performance liquid chromatography (HPLC) analysis showed that the contents of gentiocyanin derivatives were decreased in the petals of transgenic plants in comparison to those of WT plants. Ours results revealed the molecular functions of *GlaDFR1* and *GlaDFR2* in the formation of coloration, providing solid theoretical foundation and candidate genes for further genetic improvement in flower color of plants.

## 1. Introduction

The existence of natural pigments generates the colorful world of higher plants [1]. The roots, stems, leaves, flowers, fruits, and seed coats of different plants contain different types and contents of pigments, giving rise to different colors. In particular, the flower color of higher plants is mainly determined by chlorophylls, flavonoids, carotenoids, alkaloids, and betalains, with the types of accumulated pigments varying among different plants [2–8]. Under natural conditions, the anthocyanidins belonging to flavonoids exist in the form of anthocyanins, which are the glycoside derivatives. Specifically, the anthocyanidin interacts through glycosides with one or more molecules of glucose, rhamnose, galactose, or arabinose, with the hydroxyl group on the third carbon atom of the sugar ring glycosylated to generate anthocyanin [9–12]. The anthocyanin is a type of water-soluble natural pigment widely present in plants, particularly in vacuoles, determining the flower color of most plants [13]. To date, more than 550 types of anthocyanidins have been identified and categorized into at least 27 categories, while the common anthocyanidins in plants are mainly revealed in 6 categories, including pelargonidin, malvidin, cyanidin, peonidin, delphinidin, and petunidin [12–15]. These anthocyanidins are differentiated by the methylation and hydroxylation at the R1 and R2 positions of the flavonoid skeleton.

Anthocyanidins have shown a wide range of biological functions. For example, the anthocyanidins accumulated in flowers and fruits generate rich colors in plants to attract pollinators and fruit dispersers to spread pollen and fruits, respectively [16, 17], while anthocyanins accumulated in vegetative organs help plants resist biotic and abiotic stresses such as low temperature, pests, UV damage, and animal feeding [17–19]. Furthermore, the anthocyanidins are the most effective and free radical scavengers in nature for humans, showing much higher capability of scavenging free radicals than that of vitamins C and E and various healthcare functions such as antioxidation, antiaging, antimutation, anti-inflammatory, anti-infection, alleviation of cardiovascular and cerebrovascular diseases, protection of the liver, inhibition of tumor cell formation, and promotion of blood circulation [19, 20]. For example, pelargonidin has been revealed to show antithrombotic and antiplatelet activities to prevent thrombosis and improve blood circulation [21], while the anthocyanidin is also a type of natural food coloring pigment, rich in resources, safe, and nontoxic, with both nutritional and pharmacological effects [22]. Therefore, anthocyanins have shown significant prospects and applications in various medical and healthcare areas, including food, cosmetics, horticulture, and crop improvement.

In the past decades, the biosynthetic pathways of anthocyanidins have been extensively investigated and established well based on the mutants of flower color in model plants including *Antirrhinum majus*, *Arabidopsis thaliana*, *Petunia hybrida*, and *Zea mays* [15, 23, 24]. In addition to the light, temperature, abiotic stresses, and plant hormones [15, 25–38], studies have shown that the main factors involved in the biosynthetic pathway of anthocyanidins are divided into two groups: one group contains a variety of key enzymes encoded by structural genes, which are shared in plants, while the other group contains the transcriptional factors regulating the expression of structural genes and ultimately the changes in the temporal and spatial expressions of anthocyanins [15, 23, 24].

**Figure 1:**
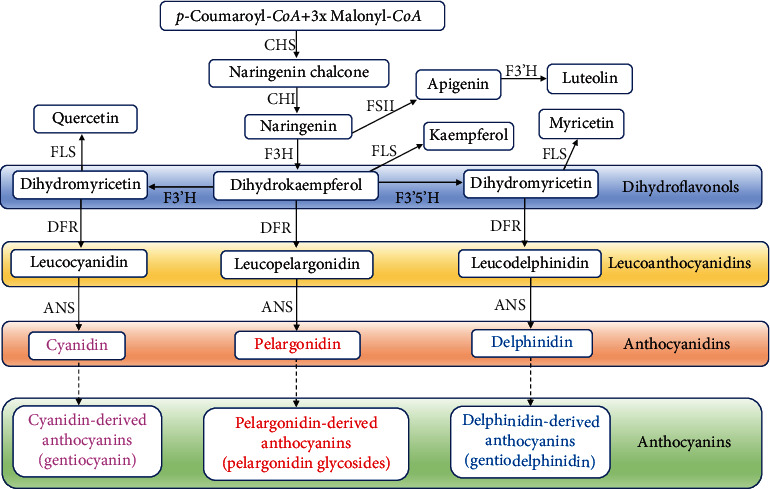
The anthocyanin biosynthesis in the flavonoid metabolic pathway in plants. Dashed lines indicate that the anthocyanidins are catalyzed by anthocyanidin glycosyltransferase (GT) and are converted into anthocyanins in the cytoplasm and vacuoles. ANS: anthocyanidin synthase; CHI: chalcone isomerase; CHS: chalcone synthase; DFR: dihydroflavonol 4-reductase; F3H: flavanone 3-hydroxylase; F3′H: flavonoid 3′-hydroxlase; F3′5′H: flavonoid 3′,5′-hydroxlase; FLS: flavonol synthase; FSII: flavone synthase.

Anthocyanidins belong to a subclass of flavonoids [13, 15, 39–41]. During the anthocyanin biosynthesis in the flavonoid metabolic pathway in plants, the chalcone synthase (CHS) is the first rate-limiting enzyme in biosynthetic pathway of anthocyanidins, catalyzing the reaction between p-coumaroyl-CoA and malonyl-CoA to generate naringenin chalcone (Figure 1). The dihydroflavonol 4-reductase (DFR) is the first key enzyme downstream in the biosynthetic pathway of anthocyanin, leading the flavonoid biosynthetic pathway to the formation of anthocyanidins, which are finally converted into anthocyanins in the cytoplasm and vacuoles [2–4, 12, 23, 42–51].

DFRs are a family of NADPH-dependent reductases encoded by a single gene or a small gene family [52–54]. For example, the *DFR* genes have been cloned from many species of plants, i.e., one copy of *DFR* gene is reported in *Vitis vinifera*, *Solanum lycopersicum*, and *Arabidopsis thaliana* [55], while two or more copies of *DFR* genes are detected in *Petunia*, *Gerbera jamesonii*, *Zea mays*, and *Lotus japonicus* [56–59]. Most of the *DFR* genes in plants are structurally composed of (1) an open reading frame (ORF) of ~1000 bp in length containing the start codon of ATG and stop codons of TAA, TGA, and TAG and (2) both 5′ and 3′ noncoding regions with largely varied lengths. These DFR genes encode about 300 to 400 stable acidic proteins [60, 61], with each DFR containing at least two typical domains, i.e., one is the NADPH binding domain and the other is the substrate-specific binding domain [2, 54].

The specificity of *DFR* gene expression varies in different tissues and organs of different plants at different developmental stages. For example, studies have shown that the expression level of *DFR* gene increases when the chrysanthemum lingual flower first appears and elongates and the petals are elongated and gradually decreases with the opening of the inflorescence [62]. In the two varieties of Asiatic lily, the *DFR* gene is highly expressed in the anthers, filaments, pistils, tepals, and red scales of the “Montreux” plant (i.e., pink tepals with spots) but only expressed in the colored scales and anthers of the “Connecticut King” plant (i.e., yellow tepals without spots), while no *DFR* gene expression is detected in the stems and leaves of both varieties. These results suggest that the *DFR* gene is only expressed in the colored floral organs showing increased expression as the flowers grow and develop, reaching the highest expression level during the flowering stage [54]. In peanuts, the *DFR* gene is only slightly expressed in the leaves and roots, moderately expressed in the petals, and highly expressed in the fruits, with the color of the seed coat highly positively correlated with the expression level of the *DFR* gene in the seed coat [63]. Although the amino acid sequences of DFR maintain high homology in many regions in different species of plants, the DFRs show varied selectivity to the three types of dihydroflavonols (i.e., DHK, DHQ, and DHM) as substrates. For example, the DFRs in *Gerbera hybrida* catalyze all three types of dihydroflavonols as substrates, while in petunia and *Cymbidium hybrids*, DFRs only effectively reduce DHQ and DHM to accumulate gentiocyanin and gentiodelphinidin in the petals without pelargonidin glycosides [12, 64–67].

The molecular mechanisms regulating the DFR substrate specificity have been investigated based on the comparative studies of amino acid sequences of DFR in petunia, corn, and snapdragon [12, 68]. The speculation that a 13-amino acid region in DFR may be responsible for DFR substrate specificity has been confirmed experimentally [12, 66, 68], indicating that the differential selection of DFR for substrates is determined by the highly conserved sequence of amino acids in the specific binding region of the substrate [12, 69]. Due to their important functions in the coloration, the DFR genes of a variety of plants have been widely applied for genetic transformation to change the flower color of a variety of transgenic plants based on various types of molecular mechanisms (Table 1) [58, 69–81]. However, studies showed that the substrate specificity of plant DFRs is not always determined by the specific sequences of amino acids, suggesting that it may not be appropriate to speculate on the function of DFRs based solely on the amino acid sequence [12, 69] due to the differential functions of *DFR* genes from the same family [69, 79]. Therefore, functional analysis of DFRs in transgenic plants with overexpression of *DFR* genes is necessary to identify the explicit functions of DFRs and the molecular mechanisms regulating the substrate specificity of DFRs [69, 79]. Furthermore, the DFR with increased activities could improve the resistance of transgenic rice to oxidation and cell death caused by bacterial diseases [82]. For example, the overexpression of *DFR* gene of *Camellia sinensis* in tobacco enhances the contents of flavonoids and antioxidant capacity [83]. Therefore, genetic engineering investigations based on *DFR* genes are of significant importance for not only changing the color of plants but also improving antioxidant activities in plants.

Studies have shown that the pelargonidin without delphinidin and cyanidin is accumulated in the orange petals of *Gentiana lutea* L. var. *aurantiaca* (M. Laínz) M. Laínz, probably with both F3H and DFR contributing to the coloration of the petals in this plant [40]. Specifically, the alteration in the substrate specificity of the DFR enzymes may be the cause for generating the pelargonidin in petals of var. *aurantiaca* [40]. In this study, we explored the molecular mechanisms regulating the DFR substrate specificity in var. *aurantiaca* based on the characterization of two cloned *DFR* genes (i.e., *GlaDFR1* and *GlaDFR2*) transformed into tobacco genome by *Agrobacterium*-mediated leaf disc method. It was speculated that the DFR derived from the *DFR* gene cloned from the petals of var. *aurantiaca* may contain the DHK substrate specificity. The phenotypic segregation was observed in the flower color of the transgenic tobacco plants with overexpression of *GlaDFR1* and *GlaDFR2*, showing darker petals than those of the wild-type (WT) plants. Our study clearly demonstrated that both GlaDFR1 and GlaDFR2 contained the functions of DFR enzymes, providing theoretical evidence for further studies on genetic improvement in flower color based on *GlaDFR1* and *GlaDFR2*.

## 2. Materials and Methods

### 2.1. Plant Materials

Previous studies have shown that the *DFR* gene is mainly expressed in petals of *Gentiana lutea* L. var. *aurantiaca*, suggesting that the change of DFR substrate specificity may be the main cause for the accumulation of pelargonidin glycosides in the petals [40]. Therefore, we chose var. *aurantiaca* as the ideal experimental materials for (1) cloning the *DFR* gene with DHK substrate specificity and determining the amino acids with DFR substrate specificity and (2) investigating the molecular mechanism regulating the synthesis and accumulation of pelargonidin in var. *aurantiaca*. Specifically, we performed (1) the bioinformatics analysis of the *GlaDFR1* and *GlaDFR2* genes and their encoded proteins and (2) characterization of the overexpression of *GlaDFR1* and *GlaDFR2* genes in transgenic tobacco plants.

Seeds of *Nicotiana tabacum* L. cv. SR1 were donated by the Biotechnology Research Institute of the Jilin Academy of Agricultural Sciences. The petals of wild-type (WT) and T1 generation of transgenic tobacco plants with overexpression of *GlaDFR1* and *GlaDFR2* were wrapped in tin foil, marked and quickly frozen in liquid nitrogen, and stored at –80°C.

### 2.2. Bacterial Strains and Plasmids

The pGEM-GlaDFR1 and pGEM-GlaDFR2 plasmids were kept at the Biological Experiment Center of Changchun Normal University, Changchun, China. The *E. coli* Trans1-T1 Phage Resistant Chemically Competent Cell and pEASY®-T1 Simple vector were purchased from Beijing Quanshijin Biotechnology Co., Ltd. (Beijing, China). *Agrobacterium tumefaciens* strain EHA105 and plant expression vector pCAMBIA1302 of 10,549 bp containing the double enzyme digestion sites of NcoI and BstEII were provided by the Biological Experiment Center of Changchun Normal University.

### 2.3. Chemical and Enzymes

The 2x TransTaq® HiFi PCR SuperMix II, 2x EasyTaq® PCR SuperMix, EasyPure® PCR Purification Kit, pEASY®-T1 Cloning Kit, EasyPure® Plasmid Miniprep Kit, EasyPure® Plant Genomic DNA Kit, EasyPure® Plant RNA Kit, Trans 15k DNA Marker, IPTG, and X-gal were purchased from Beijing Quanshijin Biotechnology Co., Ltd. (Beijing, China). Genecolour II was purchased from Beijing Jinboyi Biotechnology Co., Ltd. (Beijing, China). Ampicillin (Amp), kanamycin (Kan), cefotaxime (Cef), rifampin (Rif), hygromycin (Hyg), 1-naphthaleneacetic acid (NAA), 6-benzylaminopurine (6-BA), acetosyringone (AS), and other routinely used chemicals were purchased from Beijing Dingguo Changsheng Biotechnology Co., Ltd. (Beijing, China). T4 ligase was purchased from Dalian Bao Bioengineering Co., Ltd. (Dalian, China). Restriction endonucleases NcoI and BstEII were purchased from New English Biolabs Ltd. (Beijing, China). Hydrochloric acid, methanol, and formic acid were purchased from Beijing Dingguo Changsheng Co., Ltd. (Beijing, China).

### 2.4. Main Instruments

The main instruments used in this study included the ultralow temperature (–80°C) refrigerator (Thermo Fisher Scientific, USA), high-speed centrifuge (Sigma-Aldrich, Germany), autoclave (SX-500, TOMY Co. Ltd., Japan), ultrapure water system (Milli-Q Reference, Millipore, USA), UV-5100 spectrophotometer (Shanghai Metash Instruments Co., Ltd., Shanghai, China), ZHWY-2102 constant temperature culture oscillator (Zhicheng Co., Ltd., Shanghai, China), ZRR-5110 constant temperature dryer (Shanghai SuKe Industrial Co., Ltd., Shanghai, China), spectrophotometer (NanoDrop 2000C, Thermo Fisher Scientific, USA), automatic sample grinder (Tissuelyser-192, Shanghai Jingxin Experimental Technology, Shanghai, China), ultrasonic cleaner (KQ2200, Kunshan Ultrasonic Instruments Co., Ltd., Kunshan, China), and high-performance liquid chromatography (LC-6AD, Shimadzu, Shanghai, China).

### 2.5. Preparation of Medium and Chemical Solutions

#### 2.5.1. Medium and Chemical Solutions Used in the Cloning and PCR Amplification

The LB liquid medium contained 10 g tryptone, 10 g yeast extract, and 5 g sodium chloride, with the pH adjusted to 7.0, volumed to 1 L with double-distilled water, and sterilized with high-temperature and high-pressure steam. The LB solid medium contained 10 g tryptone, 5 g yeast extract, 10 g NaCl, and 15 g agar powder, with the pH adjusted to 7.0, volumed to 1 L with double distilled water, and sterilized with high-temperature and high-pressure steam. The 100 mg/L Amp contained 1 g ampicillin volumed to 10 mL with sterile water, filtered and sterilized with a 0.22 *μ*m filter membrane, and aliquoted and stored at –20°C. The 100 mg/L Kan contained 1 g kanamycin volumed to 10 mL with sterile water, filtered and sterilized with a 0.22 *μ*m filter membrane, and aliquoted and stored at –20°C. The 10x TBE contained 108 g Tris, 7.44 g Na_2_EDTA·2H_2_O, 55 g boric acid, added with deionized water to 1 L and mix well, stored at room temperature, and diluted to 20 times when used.

#### 2.5.2. Medium and Chemical Solutions Used in the *Agrobacterium*-Mediated Transformation of *GlaDFR1* and *GlaDFR2* into Tobacco

The YEB solid medium contained 5 g peptone, 1 g yeast extract, 0.5 g magnesium sulfate heptahydrate, 10 g sucrose, and 15 g agar, with the pH adjusted to 7.0, volumed to 1 L with ddH_2_O, and sterilized with high-temperature and high-pressure steam. The YEB solid medium contained 5 g peptone, 1 g yeast extract, 0.5 g magnesium sulfate heptahydrate, and 10 g sucrose, with the pH adjusted to 7.0, volumed to 1 L with ddH_2_O, and sterilized with high-temperature and high-pressure steam. The YEP solid medium contained 10 g peptone, 10 g yeast extract, 5 g sodium chloride, and 15 g agar, with pH adjusted to 7.2, volumed to 1 L with ddH_2_O, and sterilized with high-temperature and high-pressure steam. The YEP liquid culture medium contained 10 g peptone, 10 g yeast extract, and 5 g sodium chloride, with pH adjusted to 7.2, volumed to 1 L with ddH_2_O, and sterilized with high-temperature and high-pressure steam. The MS_0_ solid medium contained 4.43 g MS salt, 30 g sucrose, and 7.5 g agar, with pH adjusted to 5.8, volumed to 1 L with ddH_2_O, and sterilized with high-temperature and high-pressure steam. The MS solid medium was made the same as the MS_0_ solid medium with the addition of Hyg of Cef. The infection liquid medium contained 4.43 g MS salt, 30 g sucrose, and 2 g 2-(N-morpholino) ethanesulfonic acid (MES), with pH adjusted to 5.8, volumed to 1 L with ddH_2_O, sterilized with high-temperature and high-pressure steam, and added with 100 *μ*M AS after cooling to 50°C. The cocultivation solid medium contained 4.43 g MS salt, 30 g sucrose, and 7.5 g agar, with pH adjusted to 5.8, volumed to 1 L with ddH_2_O, sterilized with high-temperature and high-pressure steam, and added 0.5 mg NAA and 1 mg 6-BA after cooling to 50°C. The bacteriostatic solid medium contained 4.43 g MS salt, 30 g sucrose, and 7.5 g agar, with pH adjusted to 5.8, volumed to 1 L with ddH_2_O, sterilized with high-temperature and high-pressure steam, and added 0.5 mg NAA, 1 mg 6-BA, and 250 mg Cef after cooling to 50°C. The screening solid medium contained 4.43 g MS salt, 30 g sucrose, and 7.5 g agar, with pH adjusted to 5.8, volumed to 1 L with ddH_2_O, sterilized with high-temperature and high-pressure steam, and added 0.5 mg NAA, 1 mg 6-BA, 250 mg Cef, and 10 mg Hyg after cooling to 50°C. The rooting solid medium contained 4.43 g MS salt, 30 g sucrose, and 7.5 g agar, with pH adjusted to 5.8, volumed to 1 L with ddH_2_O, sterilized with high-temperature and high-pressure steam, and added 250 mg Cef and 10 mg Hyg after cooling to 50°C. The stock solution of Kan, Cef, Rif, and Hyg was volumed to 100 mg/mL using sterile water, filtered with 0.22 *μ*m membrane, and sterilized and aliquoted for further use (–20°C). To make the stock solution of NAA, the NAA was dissolved in pure ethanol in flask and volumed to 10 mg/mL with sterile water, filtered with 0.22 *μ*m membrane, and sterilized and aliquoted for further use (–20°C). To make the stock solution of 6-BA, the 6-BA was dissolved in 1 mol/L HCl in flask, volumed to 1 mg/mL with sterile water, filtered with 0.22 *μ*m membrane, and sterilized and aliquoted for further use (–20°C). To make the stock solution of AS, the AS was volumed to 1 mg/mL, filtered with 0.22 *μ*m membrane, and sterilized and aliquoted for further use (–20°C).

### 2.6. Bioinformatics Analysis of GlaDFR1 and GlaDFR2

#### 2.6.1. PCR Amplification of *GlaDFR1* and *GlaDFR2*

PCR amplification was performed using pGEM-GlaDFR1 and pGEM-GlaDFR2 plasmids as templates to obtain *GlaDFR1* and *GlaDFR2* genes. The 25 *μ*L PCR reaction contained 1 *μ*L template, 12.5 *μ*L 2x TransTaq® HiFi PCR SuperMix II, 9.5 *μ*L ddH_2_O, and 1 *μ*L of each primer of GlaDFRs forward and GlaDFRs reverse. Primers were designed based on the nucleotide sequences of *GlaDFR1* and *GlaDFR2* using Primer 5.0 software with the NcoI restriction site at the 5′ end of the upstream primer and the BstEII restriction site at the 5′ end of the downstream primer. The primer sequences were GlaDFRs forward 5′-ATGGATCCATGGAAGGAGGGATTTTATC-3′ and GlaDFRs reverse 5′-ACCGGGTCACCTAGTCTAGTGAATCTTG-3′ with the restriction sites underlined. The PCR procedures were as follows: predenaturation at 94°C for 3 min, followed by 35 cycles of “denaturation at 94°C for 30 sec, annealing at 56°C for 30 sec, and extension at 72°C for 1 min” and the final extension of 10 min at 72°C. The PCR products were examined with 1% agarose gel electrophoresis, recovered with AxyPrep recovery kit to collect the PCR amplification products of *GlaDFR1* and *GlaDFR2*, and purified with Easy Pure® PCR Purification Kit.

#### 2.6.2. TA Cloning of *GlaDFR1* and *GlaDFR2*

The target fragment recovered was ligated to the pEASY®-T1 Simple vector. The 5 *μ*L ligation system contained 4 *μ*L target fragment and 1 *μ*L pEASY®-T1 Simple vector. The reaction was mixed gently and placed in metal bath at 37°C for 15 min. Centrifuge tubes were placed on ice to prepare for transformation in *Escherichia coli*.

#### 2.6.3. Transformation of the Ligation Product into *Escherichia coli* Strain Trans1-T1

The ligation product was transformed into *E. coli* strain Trans1-T1 as follows. (1) The competent cells of *E. coli* strain Trans1-T1 (–80°C) were placed on ice to thaw and added 10 *μ*L of the ligation product in ice bath for 30 min. (2) The centrifuge tube was heated in water bath (42°C) for 30 sec, then quickly transferred to an ice bath for 2 min, and stood still without shaking. (3) A total of 500 *μ*L of sterile LB medium was added to the centrifuge tube, mixed well, and incubated for 1 h at 37°C and 200 rpm to revive the bacterial cells. (4) In the ultraclean workbench, the LB solid medium containing both 50 *μ*g/mL Amp and 50 *μ*g/mL Kan was added with 8 *μ*L 500 mM IPTG and 40 *μ*L 20 mg/mL X-gal, applied evenly with a sterile spreader, and left for 30 min. (5) After the IPTG and X-gal in the plates were completely absorbed, a total of 150 *μ*L bacterial solution was added, smeared evenly with a sterile spreader. The petri dishes were inverted and placed in a constant temperature incubator (37°C) overnight.

#### 2.6.4. Screening of Positive Clones

The white colonies on the culture medium were randomly selected and cultured in LB liquid medium containing 50 *μ*g/mL Amp and 50 *μ*g/mL Kan at 37°C and 200 rpm overnight. The bacterial solution was used as the template for PCR amplification. The Trans1-T1 bacterial solution was used as the negative control. The 25 *μ*L PCR reaction contained 1 *μ*L DNA template, 1 *μ*L of each of M13 forward and reverse primers, 12.5 *μ*L 2x TransTaq® HiFi PCR SuperMix II, and 9.5 ddH_2_O. The PCR procedures were as follows: predenaturation at 94°C for 3 min, followed by 35 cycles of “denaturation at 94°C for 30 sec, annealing at 56°C for 30 sec, and extension at 72°C for 1 min” and the final extension of 10 min at 72°C. The PCR products were examined using 1% agarose gel electrophoresis to detect the band of ~1.4 kb in length to confirm the successful transformation of the target gene fragment.

#### 2.6.5. Extraction and Sequencing of Positive Recombinant Plasmids

The plasmids were extracted from bacterial solution using the Easy Pure® Plasmid Miniprep Kit according to the manufacturer's protocols. The plasmids with successful transformation were verified by PCR, as described above for the screening of positive clones. The recombinant plasmids were sent to Harbin Ruibo Xingke Co., Ltd. (Harbin, China) for sequencing and identification. The successfully verified plasmids were named pEASY-GlaDFR1 and pEASY-GlaDFR2, respectively.

#### 2.6.6. Structural and Phylogenetic Analyses of GlaDFR1 and GlaDGR2

The protein primary, secondary, and tertiary structures were predicted by Protparam (http://www.expasy.org/tools/protparam.htm), SOPMA (http://npsa-pbil.ibcp.fr/cgi-bin/npsa_automat.pl?page=/NPSA/npsa_sopma.htmL), and SWISS-MODEL (http://swissmodel.expasy.org/), respectively. The Prot Scale (http://web.expasy.org/protscale/) was applied to predict the protein hydrophilicity/hydrophobicity, while TMHMM (http://www.cbs.dtu.dk/services/TMHMM/) was used to predict the protein transmembrane structure. The protein signal peptide was predicted by SignalP 4.1 Serve (http://www.cbs.dtu.dk/services/SignalP/), while the protein subcellular location was predicted by both Softberry (http://www.softberry.com/) and LOCTREE3 (http://rostlab.org). Phylogenetic relationships among a total of 20 representative amino acid sequences of DFR (13 dicots and 7 monocots) including GlaDFR1 and GlaDGR2 retrieved from the National Center for Biotechnology Information (NCBI) database (https://www.ncbi.nlm.nih.gov/) were reconstructed based on MEGA7.0 with 1000 bootstrap replicates performed [84].

### 2.7. Agrobacterium-Mediated Transformation of GlaDFR1 and GlaDFR2 into Tobacco

#### 2.7.1. Construction of Plant Overexpression Vector

To extract the pCAMBIA1302 plasmid, the *E. coli* solution containing pCAMBIA1302 plasmid kept at –80°C was activated with the LB liquid medium containing 50 mg/L Kan and cultivated overnight at 37°C with shaking at 200 rpm. A small amount of activated bacterial solution was collected with a pipette tip, which was used to draw a line on the LB solid medium containing 50 mg/L Kan. The petri dishes were inverted and placed in a constant temperature incubator (37°C), cultivated overnight until the single colonies were observed. A single colony was picked and inoculated in the LB liquid medium containing 50 mg/L Kan and cultivated overnight at 37°C with shaking at 200 rpm. The plasmids were extracted using the Easy Pure® Plasmid Miniprep Kit according to the manufacturer's protocols.

To construct the vector digestion system, each of the three plasmids pCAMBIA1302, pEASY-GlaDFR1, and pEASY-GlaDFR2 was digested with restriction enzymes NcoI and BstEII. The 120 *μ*L digestion system contained 30 *μ*L each of the three types of plasmids, 8 *μ*L of NcoI, 8 *μ*L BstEII, 16 *μ*L 10x FastDigest Green Buffer, and 58 *μ*L ddH_2_O. The reaction continued for 2 h at 37°C. After verification by agarose gel electrophoresis, the digested product with a length of ~1.1 kb (i.e., the target fragments representing *GlaDFR1* and *GlaDFR2*) was recovered with a DNA recovery kit.

To perform the linkage and transformation of target fragment and vector, T4 DNA ligase was used to ligate the recovered target gene fragments to the vector. The 10 *μ*L ligation system contained 1 *μ*L target gene fragment, 4 *μ*L vector fragment, 1 *μ*L T4 DNA ligase, 1 *μ*L 10x T4 DNA ligase buffer, and 3 *μ*L ddH2O. The ligation system was placed in a metal bath at 16°C for overnight. The two recombinant plasmids were transformed into *E. coli* strain Trans1-T1 competent cells. The successful transformation was verified by PCR. The transformed bacterial solution was spread evenly on the LB solid medium containing 50 *μ*g/mL Kan. The plates were inverted and kept in a constant temperature incubator (37°C) overnight to obtain the positive clones. A single colony was picked and inoculated in the LB liquid medium with 50 *μ*g/mL Amp and cultured overnight at 37°C and 200 rpm.

To screen the recombinant vectors, the overnight cultured bacterial broth (1 *μ*L) was used as a template for PCR verification with the Trans1-T1 broth as the negative control. The 25 *μ*L reaction system contained 1 *μ*L template, 1 *μ*L of each of primers *GlaDFRs* forward and *GlaDFRs* reverse, 12.5 *μ*L 2x EasyTaq® PCR SuperMix, and 9.5 *μ*L ddH_2_O. The reaction procedures were as follows: predenaturation at 94°C for 3 min, followed by 35 cycles of “denaturation at 94°C for 30 sec, annealing at 56°C for 30 sec, and extension at 72°C for 1 min” with the final extension of 10 min. The PCR products were examined with the agarose gel electrophoresis. The successful ligation of the target genes into the pCAMBIA1302 vector was verified by the presence of PCR product of ~1.1 kb in length.

The successful construction of recombinant plasmids was further confirmed by plasmid PCR verification and restriction digestion verification based on endonucleases NcoI and BstEII. The PCR and digestion products were examined with the agarose gel electrophoresis to verify the successful transformation based on the band size of ~1.1 kb. The successfully verified recombinant plasmids were kept at –20°C and used to transform into *Agrobacterium tumefaciens* EHA105.

#### 2.7.2. Preparation of *Agrobacterium tumefaciens* EHA105 Competent Strain

A small amount of bacterial solution of *Agrobacterium tumefaciens* strain EHA105 kept at –80°C was collected with a pipette dip to streak on the YEB solid medium containing 50 *μ*g/mL Rif. The petri dishes were inverted and placed in a constant temperature incubator (28°C) for ~36 h. A single colony was picked and inoculated in 3 mL YEB liquid medium containing 50 *μ*g/mL Rif and incubated at 28°C and 200 rpm for ~24 h. The activated bacterial solution (1 mL) was inoculated into 50 mL YEB liquid medium, cultured at 28°C with shaking at 200 rpm until the OD_600_ value reached 0.6–0.8. The bacterial solution was transferred into a 50 mL centrifuge tube, kept in the ice bath for 30 min, and shaken occasionally to facilitate the bacteria enter a dormant state. Then, the bacterial solution was centrifuged at 5000 rpm for 10 min at 4°C to discard the supernatant and keep the bacterial pellet, which was resuspended with 1 mL of 20 mM CaCl_2_ precooled on ice and added with glycerol to form *Agrobacterium* competent cells. The competent cells were aliquoted, quick-frozen in liquid nitrogen, and kept at –80°C.

#### 2.7.3. Transformation of *Agrobacterium tumefaciens* Strain EHA105 by Liquid Nitrogen Freezing and Thawing Method

A total of 800 *μ*L of YEP liquid medium was added to a sterilized 1.5 mL centrifuge tube and preheated at 28°C. A total of 100 *μ*L of *Agrobacterium* EHA105 competent cells thawed on ice was added with a total of 6 *μ*L of the plasmid to be transformed, mixed slightly and kept on ice bath for 30 min, then placed in liquid nitrogen for 5 min, added to preheated YEP liquid medium at 28°C, and cultivated at 200 rpm until the OD_600_ value reached 0.6–0.8. A total of 200 *μ*L of the activated bacterial solution was inoculated on the YEP solid medium containing 50 *μ*g/mL Rif and 50 *μ*g/mL Kan. The petri dishes were inverted and cultured at 28°C for 24-48 h until the single colonies were observed. A single colony was picked and inoculated in 5 mL YEP liquid medium containing 50 *μ*g/mL Rif and 50 *μ*g/mL Kan and cultured for 24–48 h at 28°C with shaking at 200 rpm. The plasmid PCR verification was performed to confirm the successful transformation of pCAMBIA1302-GlaDFR1 and pCAMBIA1302-GlaDFR2 into *Agrobacterium* cells. The 25 *μ*L reaction system contained 1 *μ*L template, 1 *μ*L of each of the *GlaDFRs* forward and *GlaDFRs* reverse primers, 12.5 *μ*L 2x EasyTaq® PCR SuperMix, and 9.5 *μ*L ddH_2_O. The PCR procedures were as follows: predenaturation at 94°C for 3 min, followed by 35 cycles of “denaturation at 94°C for 30 sec, annealing at 56°C for 30 sec, and extension at 72°C for 1 min” with the final extension of 10 min. The PCR products were examined with agarose gel electrophoresis. The bacterial solution containing the positive plasmid was added with an appropriate amount of glycerin and kept in the ultralow temperature refrigerator.

#### 2.7.4. Preparation of Tobacco Materials

Tobacco seeds with full grains, uniform size, and healthy colors were selected and soaked in H_2_O_2_ solution (10%) in 2 mL centrifuge tube for 5 min, slowly shaking to make the seeds fully contact the solution. A pipette was used to carefully suck up the H_2_O_2_ solution and add 1 mL of sterile water to wash the seeds twice. The soaking of seeds with H_2_O_2_ solution and washing of seeds with sterile water were repeated thrice. The sterilized tobacco seeds were evenly spread on the sterilized filter paper with a pipette tip, placed in a sterilized petri dish, wrapped with a sealing film, and transferred to a growth chamber with constant temperature (25°C) and a photoperiod cycle of 16 h light and 8 h dark. After the cotyledons were completely unfolded, the seedlings were transferred to a culture flask containing MS_0_ solid medium to continue to grow.

#### 2.7.5. Preparation of Bacterial Solution

The *Agrobacterium* strain EHA105 containing the vectors pCAMBIA1302-GlaDFR1 and pCAMBIA1302-GlaDFR2 kept at –80°C was used to streak on YEP solid medium containing 50 *μ*g/mL Kan and 50 *μ*g/mL Rif and cultivated at 28°C for 48 h. A single colony was picked and inoculated in the YEP liquid medium containing 50 *μ*g/mL Kan and 50 *μ*g/mL Rif and cultured overnight at 28°C with shaking at 200 rpm. The bacterial solution (1 mL) was inoculated in 50 mL of YEP liquid medium containing 50 *μ*g/mL Kan and 50 *μ*g/mL Rif and cultured overnight at 28°C with shaking at 200 rpm. The AS solution with a final concentration of 100 *μ*M was added to the culture in the ultraclean workbench and incubated for 30 min. The *Agrobacterium* cultural solution was centrifuged for 10 min at 4°C and 5000 rpm. The supernatant was discarded, and the bacteria were resuspended in the infection liquid medium containing 100 *μ*M AS. The *Agrobacterium* solution was diluted to obtain the OD_600_ value of 0.6–0.8 and kept at room temperature.

#### 2.7.6. *Agrobacterium*-Mediated Genetic Transformation and the Acquisition of Resistant Seedlings of Tobacco Plants

To prepare the explants, the sterile tobacco leaves of 4–5 cm in width were selected in the ultraclean workbench and placed on the filter paper soaked in sterile water, with the edges and main veins carefully removed with a scalpel to make the explants of 5 mm × 5 mm in size for use. The prepared explants were soaked for 20 min in the *Agrobacterium* suspension containing pCAMBIA1302-GlaDFR1 and pCAMBIA1302-GlaDFR2 plasmids and shaken several times. The explants were placed on sterile filter paper to dry the *Agrobacterium* bacterial liquid, transferred to the cocultivation medium with the back of the leaf contacting the medium, sealed with the sealing film, and incubated in dark at 28°C for 3 days. After the cocultivation, the explants were washed 3 or 4 times with MS_0_ liquid medium containing 250 mg/L Cef until the liquid became clear. The rinsed explants were placed on the sterile filter paper to dry the liquid, transferred to a bacteriostatic medium, and cultured for 7 days at a constant temperature (25°C) with a photoperiod cycle of 16 h light and 8 h dark. After the bacteriostatic culture, the explants were transferred to the screening medium and screened twice. The first screening of ~20 days was performed on the screening medium containing Hyg of 10 *μ*g/mL, while the second screening on the medium containing Hyg of 15 *μ*g/mL with adventitious buds observed. The adventitious buds with 3 or 4 leaves were cut off with a scalpel and transferred to a culture flask containing rooting medium to continue culturing. When the resistant plants in the culture flasks were 8–10 cm in height and the root longer than 5 cm, the culture flasks were opened to refine the seedlings for 3 days and then the seedlings were transplanted to the soil to grow in the greenhouse.

#### 2.7.7. Transgenic Tobacco Plants with Overexpression of *GlaDFR1* and *GlaDFR2*

The resistant tobacco plants were obtained through Hyg resistance screening. Both WT and resistant tobacco DNA were extracted using the EasyPure® Plant Genomic DNA Kit based on the manufacturer's protocols and used as templates to perform the PCR verification to further verify the integration of the target genes in order to obtain the positive transgenic tobacco plants. The ddH_2_O and WT tobacco plants were used as blank and negative controls, respectively. The 25 *μ*L PCR reaction contained 1 *μ*L template, 1 *μ*L of each of the *GlaDFRs* forward and *GlaDFRs* reverse primers, 12.5 *μ*L 2x EasyTaq® PCR SuperMix, and 9.5 *μ*L ddH_2_O. The PCR procedures were as follows: predenaturation at 94°C for 3 min, followed by 35 cycles of “denaturation at 94°C for 30 sec, annealing at 56°C for 30 sec, and extension at 72°C for 1 min” with the final extension of 10 min. The PCR products were examined with the agarose gel electrophoresis. The positive transgenic plants were kept to continue to grow until the seeds were harvested.

### 2.8. Genetic and Phenotypic Analysis of Transgenic Tobacco Plants with Overexpression of GlaDFR1 and GlaDFR2

#### 2.8.1. Screening and PCR Verification of T1 Generation of Transgenic Tobacco Plants

The T1 generation of transgenic tobacco plants with overexpression of *GlaDFR1* and *GlaDFR2* was screened based on the Hyg resistance screening. Seeds of WT and T1 generation of transgenic tobacco plants with overexpression of *GlaDFR1* and *GlaDFR2* were disinfected and sterilized. Then, the sterile seeds of WT were sown into a petri dish of MS_0_ solid medium, while the seeds of T1 generation transgenic tobacco plants were sown into a petri dish of MS_0_ solid medium containing 50 *μ*g/mL Hyg. The petri dishes were wrapped with parafilm and placed in the culture rack. After the seeds germinated, the ratio of the seedlings with hygromycin resistance growing normally and the seedlings without hygromycin resistance becoming albino was calculated. PCR verification of T1 generation of transgenic tobacco with overexpression of *GlaDFR1* and *GlaDFR2* was performed following the same procedure as that for the PCR verification of T0 generation transgenic tobacco plants.

We further performed the semiquantitative real-time PCR analysis to determine the quantitative expression of *GlaDFR1* and *GlaDFR2* in the transgenic tobacco plants. Total RNA of the transgenic tobacco was extracted using the EasyPure® Plant RNA Kits based on the manufacturer's protocols. The concentration of total RNA was determined, and the RNA samples were stored at –80°C.

The TransScript® One-Step gDNA Removal and cDNA Synthesis SuperMix Reverse Transcription Kits were used to synthesize the first strand of cDNA using the total RNA as template. The 20 *μ*L reaction system contained 1 *μ*L RNA template, 10 *μ*L 2x TS Reaction Mix, 1 *μ*L Anchored Oligo(dT)_18_ Primer, 1 *μ*L TransScript® RT/RI Enzyme Mix, 1 *μ*L gDNA Removal, and 6 *μ*L RNase-free water. The reaction procedures were as follows: RNA template, Anchored Oligo(dT)18 Primer, and RNase-free water were added to the centrifuge tube, mixed and incubated in a metal bath at 65°C for 5 min and ice bath for 2 min, then added other reaction components to the system, mixed gently, incubated at 42°C for 30 min and then heated at 85°C for 5 sec to inactivate the TransScript® RT/RI and gDNA removal. The 25 *μ*L PCR reaction system contained 1 *μ*L cDNA template, 1 *μ*L of each of the PB-GlaDFRs forward and PB-GlaDFRs reverse primers, 12.5 *μ*L 2x EasyTaq® PCR SuperMix, and 9.5 *μ*L nuclease-free water. The WT tobacco was used as a negative control. The reaction procedures were as follows: predenaturation at 94°C for 3 min, followed by 35 cycles of “denaturation at 94°C for 30 sec, annealing at 56°C for 30 sec, and extension at 72°C for 1 min” with the final extension of 10 min.

#### 2.8.2. Observation of the Petal Phenotype of Transgenic Tobacco Plants with Overexpression of *GlaDFR1* and *GlaDFR2*

The T1 transgenic tobacco plants detected with high expression by real-time PCR were allowed to grow until flowering stage. The petal color of the transgenic tobacco plants was observed and compared with that of WT tobacco plants. The lines with darkened petals were recorded with the petals collected, wrapped in tin foil, and frozen in liquid nitrogen.

#### 2.8.3. Determination of Anthocyanins and Flavonols in the Petals of Transgenic Tobacco with Overexpression of *GlaDFR1* and *GlaDFR2*

To extract the anthocyanins from tobacco petals, the mortar, spoon, and centrifuge tube were precooled with liquid nitrogen. The petal samples stored at –80°C were placed into a mortar, added with liquid nitrogen, quickly ground into powder. Then, 0.3 g of the sample was quickly and accurately weighed and transferred into a centrifuge tube and added with 1 mL of anthocyanin extraction solution. The centrifuge tube was wrapped in tin foil and placed at 4°C to avoid light for 12 h. Then, the extraction solution (hydrochloric acid, methanol, and ddH_2_O prepared in a volume ratio of 1 : 24 : 75) was centrifuged at 4°C and 10,000 rpm for 10 min. The supernatant was collected, filtered through a 0.22 *μ*m nylon microporous filter, and stored the filtrate at 4°C in the dark for later analyses of high-performance liquid chromatography (HPLC) and high-performance liquid chromatography-mass spectrometry (HPLC-MS). To detect the anthocyanins based on HPLC analysis, the Acchrom XUnion C18 chromatographic column was used with the mobile phase A of 5% formic acid and the mobile phase B of methanol. The column oven temperature was set to 35°C. The flow rate was set to 1 mL/min and the detection wavelength set to 520 nm for anthocyanins and 360 nm for flavonols.

## 3. Results and Discussion

### 3.1. TA Cloning and Verification of GlaDFR1 and GlaDFR2

**Figure 2:**
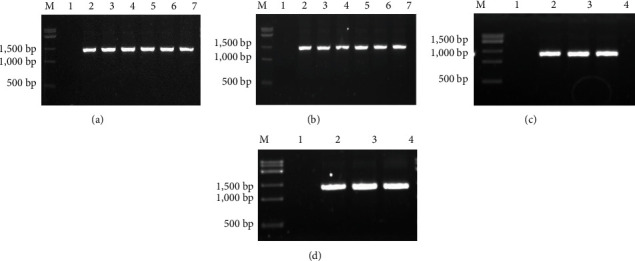
Verification of TA cloning of genes *GlaDFR1* and *GlaDFR2* based on bacterial liquid PCR of (a) pEASY-GlaDFR1 and (b) pEASY-GlaDFR2 and plasmid PCR of (c) pEASY-GlaDFR1 and (d) pEASY-GlaDFR2. Lane M represents the 15K DNA marker. Lane 1 in (a, b) represents the negative control based on *E. coli* strain Trans1-T1. Lane 1 in (c, d) represents the negative control based on ddH_2_O. Lanes 2–7 in (a, b) represent the bacterial liquid PCR products (~1.4 kb). Lanes 2–4 in (c, d) represent the plasmid PCR products (~1.4 kb).

As verified by bacterial liquid and plasmid PCR, the bacterial transformation of cloned genes *GlaDFR1* and *GlaDFR2* was successful (Figure 2).

### 3.2. Bioinformatics Analysis of GlaDFR1 and GlaDFR2

**Figure 3:**
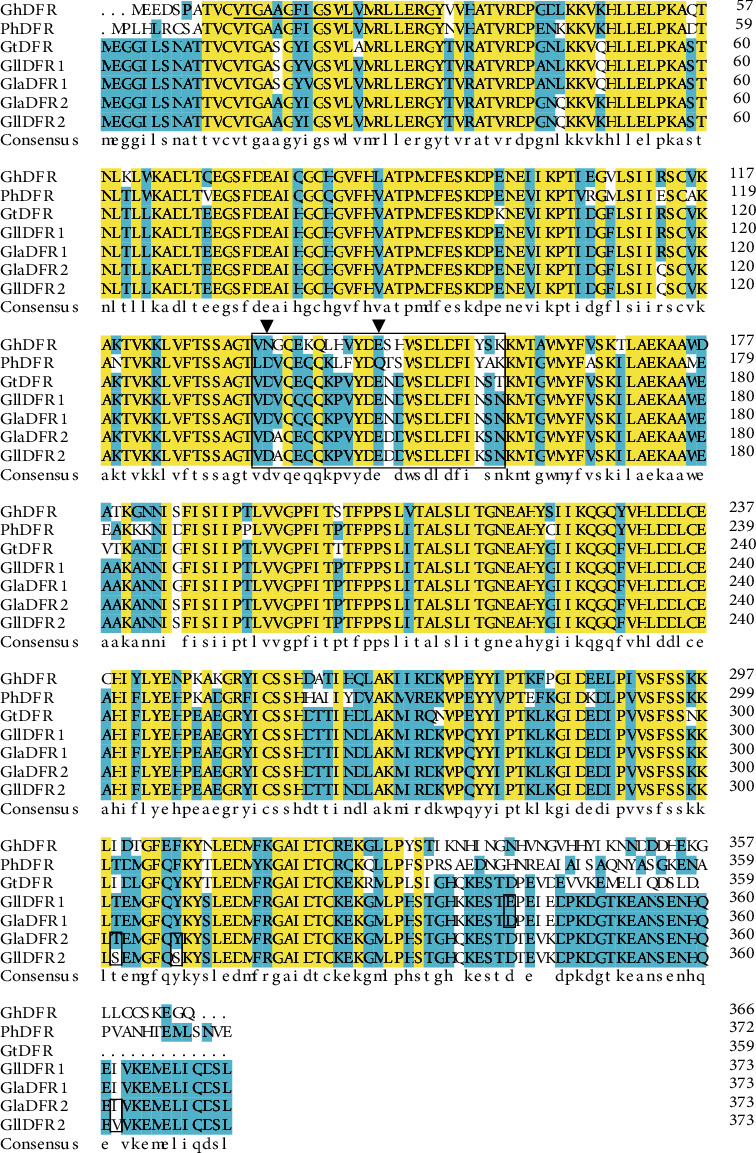
Comparative analysis of 7 amino acid sequences of *Gerbera hybrid* (GhDFR, CAA78930), *Petunia hybrida* (PhDFR, AAF60298), *Gentiana triflora* (GtDFR, BAA12736), *G. lutea* L. var. *lutea* (GllDFR1, ATP60542; GllDFR2, ATP60540), and *G. lutea* L. var. *aurantiaca* (GlaDFR1, ATP60543; GlaDFR2, ATP60541). Asterisks represent the stop codons. The NADPH binding site contains a total of 21 amino acid residues underlined at the N-terminus. The asparagine (N) and glutamine (Q) at positions 134 and 145 are indicated with arrowheads for *Gerbera hybrid* and *Petunia hybrid*, respectively. The substrate specific binding site containing a total of 26 amino acids is indicated by the large box. The different amino acids between var. *lutea* and var. *aurantiaca* are indicated by four small boxes.

The results of the comparative analysis of the amino acid sequences encoded by *GlaDFR1* and *GlaDFR2* genes and those of *Gentiana triflora*, *Gerbera hybrid*, and *Petunia hybrida* are shown in Figure 3. Both GlaDFR1 and GlaDFR2 proteins belonged to the NADPH-dependent short chain reductase family, each containing a NADPH binding site of 21 amino acid residues at the N-terminus and a substrate-specific binding site of 26 amino acids.

The type of substrate of DFR is determined by the specificity of the amino acid sequence in the substrate binding region. Specifically, based on the type of amino acid at position 134 in the substrate binding region, DFRs are categorized into three types, including Asn-type, Asp-type, and Asn/Asp-type, with Asn-type as the most common type of DFR, while Asp-type and Asn/Asp-type DFRs found in several species of plants, suggesting that Asp-type and Asn/Asp-type DFRs were evolved from Asn-type DFR [61]. Our results of comparative analysis of amino acid sequences showed that both GlaDFR1 and GlaDFR2 were Asp-type DFRs.

Although the DFR gene has been cloned from a variety of plants, the 3D structure of the DFR protein is rarely studied, and the enzymes and their molecular mechanism determining the specificity of the DFR substrate remain unknown. Studies have revealed a total of 8 gene clusters encoding DFR-like proteins in *Freesia hybrida* [85]. By constructing a prokaryotic expression vector for three of the *FhDFR* genes, the *DFR* gene was expressed in *E. coli*. The results of enzymatic activity analysis based on complementary experiments using *Arabidopsis* dfr (tt3-1) mutants showed that FhDFR1, FhDFR2, and FhDFR3 transformed DHM to produce leucodelphinidin, while FhDFR2 also catalyzed the conversion of DHQ to leucocyanidin, but FhDFR1, FhDFR2, and FhDFR3 could not use DHK as a substrate [85]. These results were consistent with those reported previously, showing the same types of anthocyanins accumulated in the petals of *Freesia* (i.e., gentiocyanin and gentiodelphinidin) [12]. Similarly, the substrate-specific binding site containing a total of 26 amino acids was identified in both GlaDFR1 and GlaDFR2 (Figure 3), suggesting the shared functions of both GlaDFR1 and GlaDFR2 in the biosynthesis of anthocyanidins.

**Figure 4:**
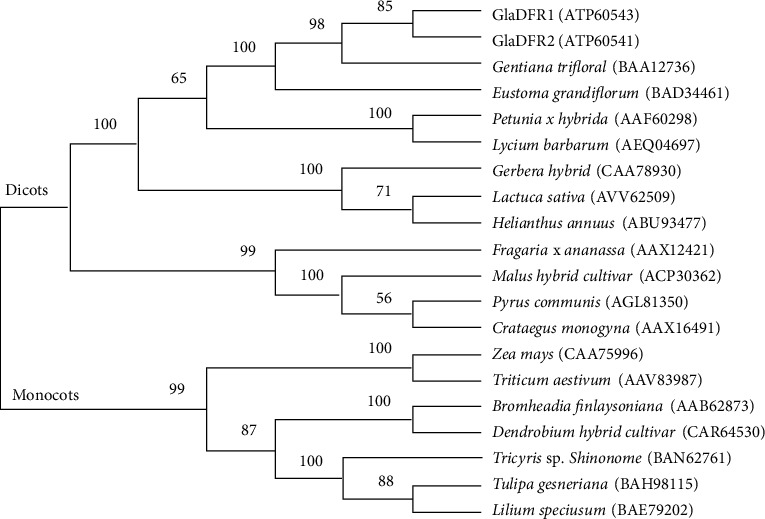
Phylogenetic relationships among a total of 20 amino acid sequences of DFR (7 monocots and 13 dicots). GenBank accession numbers are given in the parentheses. Bootstrap support (%) based on 1000 replicates is given next to the branches. GlaDFR1 and GlaDFR2 are derived from *Gentiana lutea* var. *aurantiaca.*

Phylogenetic relationships among a total of 20 amino acid sequences of DFR were reconstructed based on MEGA7.0 (Figure 4). The results showed that DFRs of monocots and dicots were revealed in two monophyletic groups. The amino acid sequences of GlaDFR1 and GlaDFR2 were closely related to those of *Gentian triflora* and *Eustoma grandiflorum*, allied in one monophyletic group strongly supported with high bootstrap value of 100. The topology of the phylogenetic tree clearly revealed the divergence of DFRs in dicots after the separation between monocots and dicots.

### 3.3. Structural Analysis of GlaDFR1 and GlaDFR2

The primary structure of GlaDFR1 and GlaDFR2 proteins was predicted based on Protparam (Table 1). The results showed that both GlaDFR1 and GlaDFR2 were stable proteins based on their instability coefficient of less than 40. Furthermore, both GlaDFR1 and GlaDFR2 were hydrophilic proteins based on the ratio of the total hydrophilic value of all amino acids to the total number of amino acids, while the values of the isoelectric point indicated that both GlaDFR1 and GlaDFR2 were acidic proteins.

**Figure 5:**
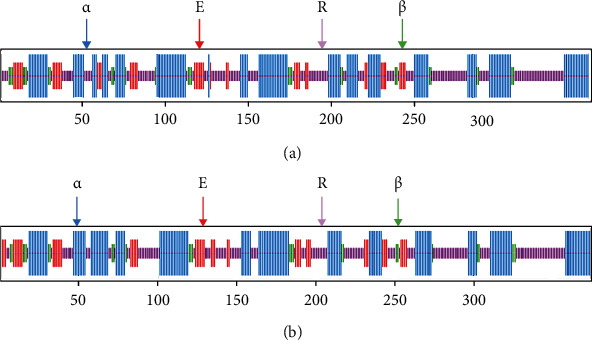
Secondary structure of both (a) GlaDFR1 and (b) GlaDFR2 proteins with a total of 374 amino acids predicted based on SOPMA showing the *α*-helix (*α*), extended strand (E), random coil (R), and *β*-turn (*β*) in blue, red, pink, and green zones, respectively.

The secondary structure of both GlaDFR1 and GlaDFR2 proteins was predicted based on SOPMA (Figure 5). Results showed that among the 374 amino acids in both GlaDFR1 and GlaDFR2, the secondary structure was mainly composed of *α*-helix and random coils, accounting for 38.24% and 41.98% in GlaDFR1 and 38.77% and 41.18% in GlaDFR2, respectively. The *β*-turns accounted for 7.49% and 6.68% and extended strands accounted for 12.30% and 13.37% in GlaDFR1 and GlaDFR2 proteins, respectively.

**Figure 6:**
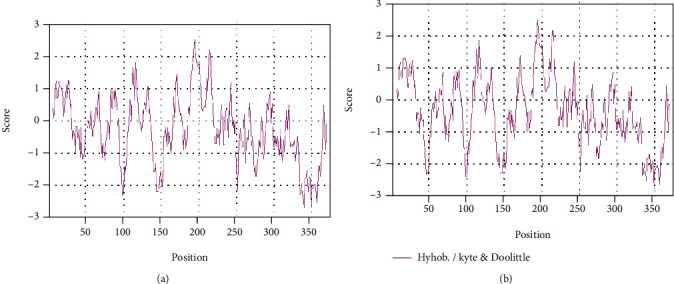
The hydrophilicity/hydrophobicity analysis of (a) GlaDFR1 and (b) GlaDFR2 proteins based on Prot Scale.

The hydrophilicity/hydrophobicity of GlaDFR1 and GlaDFR2 proteins was predicted by Prot Scale (Figure 6). Results showed that the maximum values of ratio hydrophilicity/hydrophobicity of GlaDFR1 and GlaDFR2 proteins were 2.511 and 2.511, while the minimum values were –2.733 and –2.767, respectively. In the entire peptide chains, there were more hydrophilic amino acid residues than hydrophobic ones, indicating that both GlaDFR1 and GlaDFR2 were hydrophilic proteins. These results were consistent with those based on the primary structure of these two proteins.

**Figure 7:**
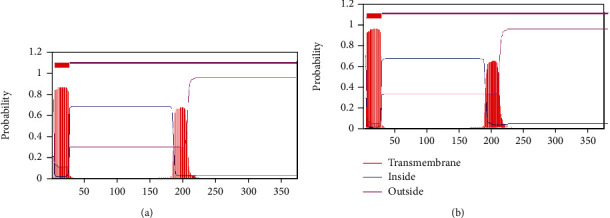
The transmembrane structure of (a) GlaDFR1 and (b) GlaDFR2 proteins predicted based on TMHMM with the transmembrane regions indicated in the hydrophobic region at the amino acid positions of 5 to 27.

The transmembrane structure of GlaDFR1 and GlaDFR2 proteins was predicted based on TMHMM (Figure 7). Results showed that both GlaDFR1 and GlaDFR2 proteins contained a transmembrane region at the amino acid positions of 5 to 27, located in the hydrophobic region, indicating that GlaDFR1 and GlaDFR2 proteins functioned as either (1) membrane receptors or (2) ion channel proteins or anchor proteins.

**Figure 8:**
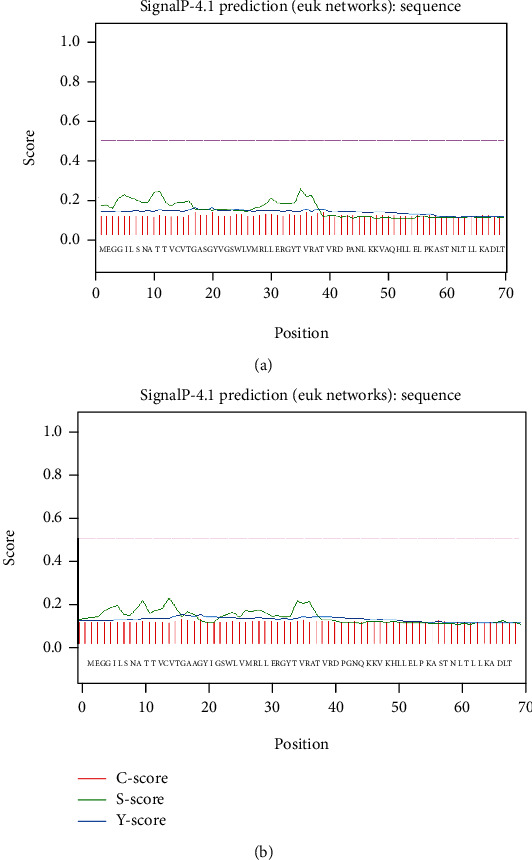
The absence of signal peptides in GlaDFR1 and GlaDFR2 proteins based on the SignalP 4.1 Server shown in the first 70 amino acids out of the entire length of 370 amino acids of both proteins.

The secretion of the nascent peptide chains out of cells is determined by the signal peptide. Therefore, we predicted the signal peptides of GlaDFR1 and GlaDFR2 proteins based on the SignalP 4.1 Server (Figure 8). Results showed that neither GlaDFR1 nor GlaDFR2 proteins contained any signal peptides. Therefore, these two proteins were not secretory proteins and cannot participate in transmembrane transport.

**Figure 9:**
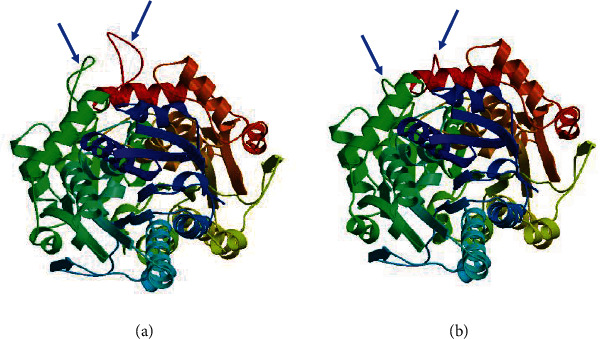
Tertiary structure of (a) GlaDFR1 and (b) GlaDFR2 proteins predicted based on SWISS-MODEL. Arrows indicate approximately the structural differences between GlaDFR1 and GlaDFR2 proteins.

The tertiary structure of both GlaDFR1 and GlaDFR2 proteins was predicted based on SWISS-MODEL (Figure 9). Results showed that the tertiary structures of GlaDFR1 and GlaDFR2 proteins were largely congruent.

To further investigate the molecular mechanisms regulating the substrate specificity of DFR, we continued to transform both *GlaDFR1* and *GlaDFR2* into tobacco genome and to characterize their expressions at transcriptional and phenotypic levels.

### 3.4. Agrobacterium-Mediated Transformation of GlaDFR1 and GlaDFR2 into Tobacco

To further investigate the function of *DFR* genes in the anthocyanidin synthesis of *Gentiana lutea* L. var. *aurantiaca*, we constructed the overexpression vectors pCAMBIA1302-GlaDFR1 and pCAMBIA1302-GlaDFR2 regulated by the promoter CaMV 35S using the *Agrobacterium*-mediated leaf disc transformation of tobacco. The resistant plants were selected based on the explants cultured on the medium containing Hyg. The T1 transgenic tobacco plants with overexpression of *GlaDFR1* and *GlaDFR2* were obtained by selfing and molecular verification.

#### 3.4.1. Verification of Recombinant Plant Expression Vector

**Figure 10:**
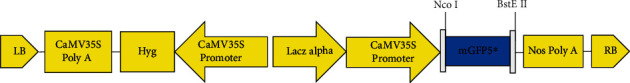
Structure of T-DNA showing the recombinant plant overexpression vectors (pCAMBIA1302-GlaDFR1 and pCAMBIA1302-GlaDFR2) constructed based on the replacement of *GFP* gene (digested with NcoI and BstEII restriction endonucleases) by *GlaDFR1* and *GlaDFR2*, respectively. LB: left border; RB: right border; Hyg: hygromycin resistance selection gene.

The verified plasmids pEASY-GlaDFR1 and pEASY-GlaDFR2 based on sequencing and the binary expression vector pCAMBIA1302 were digested with NcoI and BstEII restriction endonucleases. The digested products of *GlaDFR1* and *GlaDFR2* were recovered, purified, and used to replace the *GFP* gene on the pCAMBIA1302 vector to obtain the final overexpression vectors pCAMBIA1302-GlaDFR1 and pCAMBIA1302-GlaDFR2 (Figure 10).

**Figure 11:**
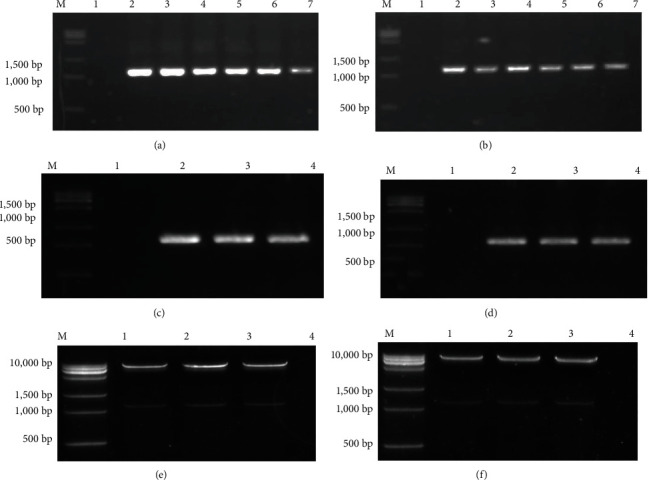
The recombinant plant overexpression vectors (pCAMBIA1302-GlaDFR1 and pCAMBIA1302-GlaDFR2) transformed into *E. coli* strain Trans1-T1 confirmed by the bacterial liquid PCR based on (a) pCAMBIA1302-GlaDFR1 and (b) pCAMBIA1302-GlaDFR2, the plasmid PCR based on (c) pCAMBIA1302-GlaDFR1 and (d) pCAMBIA1302-GlaDFR2, and the double enzyme digestion (i.e., NcoI and BstEII) of (e) pCAMBIA1302-GlaDFR1 and (f) pCAMBIA1302-GlaDFR2. Lane M represents the 15K DNA marker. Lane 1 in (a, b) represents the negative control based on *E. coli* strain Trans1-T1. Lane 1 in (c, d) represents the negative control based on ddH_2_O. Lanes 2–7 in (a, b) represent the bacterial liquid PCR products (~1.1 kb). Lanes 2–4 in (c, d) represent the plasmid PCR products (~1.1 kb). Lanes 1–3 in (e, f) represent the double enzyme digestion products of the plasmids containing the target genes (~10 kb and~1.1 kb).

The two recombinant plant expression vectors were transformed into *E. coli* strain Trans1-T1, respectively, and confirmed by the bacterial liquid PCR and plasmid PCR verification, showing the expected band of ~1.1 kb in size of the target genes. Results of double enzyme digestion verification revealed a band of ~1.1 kb and a linear vector band (~10 kb) matching the expected sizes, indicating the successful construction of plant expression vectors pCAMBIA1302-GlaDFR1 and pCAMBIA1302-GlaDFR2 (Figure 11).

#### 3.4.2. Bacterial *GlaDFR1* and *GlaDFR2* Gene Expression Vectors

**Figure 12:**
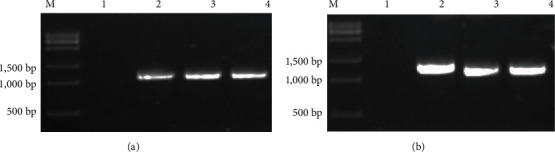
The recombinant plant overexpression vectors (a) pCAMBIA1302-GlaDFR1 and (b) pCAMBIA1302-GlaDFR2 transformed into *Agrobacterium tumefaciens* strain EHA105 confirmed by the plasmid PCR. Lane M represents the 15K DNA marker. Lane 1 represents the negative control based on ddH_2_O. Lanes 2–4 represent the plasmid PCR products (~1.1 kb).

The recombinant vectors pCAMBIA1302-GlaDFR1 and pCAMBIA1302-GlaDFR2 were transformed into *Agrobacterium tumefaciens* strain EHA105 competent cells by freezing and thawing method. The results of plasmid PCR verification revealed successful transformation of the recombinant vectors into the *Agrobacterium* EHA105 competent cells (Figure 12). These vectors were used for the genetic transformation of tobacco plants.

#### 3.4.3. Resistant Tobacco Plants with Overexpression of *GlaDFR1* and *GlaDFR2*

As one of the antibiotics used to effectively inhibit the growth of *Agrobacterium tumefaciens* with less inhibitory effect on the growth and differentiation of the recipient plants, Cef was applied in our study to obtain the optimal antibacterial and regeneration effects and high seedling rate in tobacco. These results were consistent with those reported previously [86]. Hyg was applied as the screening agent in the transformation to ensure that the transformed cells could grow and differentiate, while inhibiting the growth of untransformed cells. Our preliminary experiments showed that the callus of tobacco was sensitive to Hyg, with the leaf discs of tobacco growing rapidly on the medium without Hyg. As the concentration of Hyg in the medium increased, the growth of the leaf discs was inhibited. When the concentration of Hyg was set to 10 mg/L, the growth of callus was evidently inhibited, showing the growth rate of 50%. Therefore, Hyg with a concentration of 10 mg/L was selected for preliminary screening of tobacco callus, and then, the concentration was increased to 15 mg/L for second round of screening to obtain the resistant tobacco lines with overexpression of *GlaDFR1* and *GlaDFR2*.

**Figure 13:**
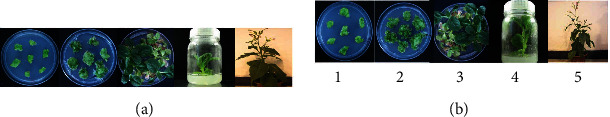
Resistant tobacco plants with overexpression of (a) *GlaDFR1* and (b) *GlaDFR2* at different developmental stages: 1: leaf disc on the cocultivation medium; 2 and 3: resistant seedlings selected on the screening medium; 4: resistant seedlings on the rooting medium; 5: flowering transgenic tobacco plants.

With the two recombinant plasmids pCAMBIA1302-GlaDFR1 and pCAMBIA1302-GlaDFR2 transformed into *Agrobacterium tumefaciens* strain EHA105 by freezing and thawing method, the leaf disc method of genetic transformation mediated by *Agrobacterium tumefaciens* was used to successfully obtain the resistant tobacco plants with overexpression of *GlaDFR1* and *GlaDFR2*. A total of 31 and 35 independent resistant tobacco lines were obtained out of ~300 explants, with the transformation rates of 10.3% and 11.7% for *GlaDFR1* and *GlaDFR2*, respectively (Figure 13).

#### 3.4.4. PCR Verification of T0 Generation of Transgenic Tobacco Plants with Overexpression of *GlaDFR1* and *GlaDFR2*

**Figure 14:**
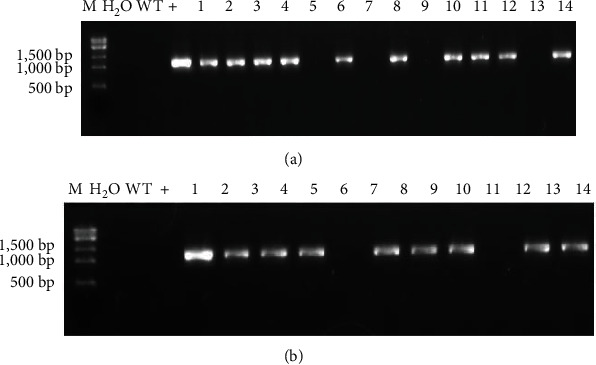
PCR verification of T0 generation of transgenic tobacco plants with overexpression of (a) *GlaDFR1* and (b) *GlaDFR2*. Lane M represents the 15K DNA marker. WT: wild type. Lane “+” represents the vector pCAMBIA1302 containing the target genes (i.e., *GlaDFR1* and *GlaDFR2*). Lanes 1–13 represent some of the tobacco plants containing the target gene (~1.1 kb) with blank lanes representing the nontransgenic tobacco plants. Lane “H_2_O” represents the negative control based on ddH_2_O.

In order to verify the successful integration of the target gene fragments into the genome of the tobacco plants, the total DNA was extracted from the leaves of both WT and hygromycin-resistant tobacco plants and used as templates to perform PCR verification on the T0 resistant tobacco plants transformed with pCAMBIA1302-GlaDFR1 and pCAMBIA1302-GlaDFR2 (Figure 14). Results showed that the *GlaDFR1* and *GlaDFR2* genes were detected in 17 and 19 transgenic tobacco plants with overexpression of *GlaDFR1* and *GlaDFR2*, respectively. The transgenic lines of tobacco plants were transferred to the greenhouse to generate T1 generation used for investigating the genetic and phenotypic stability of *GlaDFR1* and *GlaDFR2*.

### 3.5. Genetic and Phenotypic Analysis of Transgenic Tobacco Plants with Overexpression of GlaDFR1 and GlaDFR2

With many factors affecting the genetic stability, the maintenance of genetic and phenotypic stability of the target genes is crucial in transgenic breeding [87, 88]. Therefore, we further performed genetic and phenotypic analysis of transgenic tobacco plants with overexpression of *GlaDFR1* and *GlaDFR2.*

#### 3.5.1. Hygromycin Resistance Screening and PCR Verification of T1 Generation of Transgenic Tobacco Seeds with Overexpression of *GlaDFR1* and *GlaDFR2*

**Figure 15:**
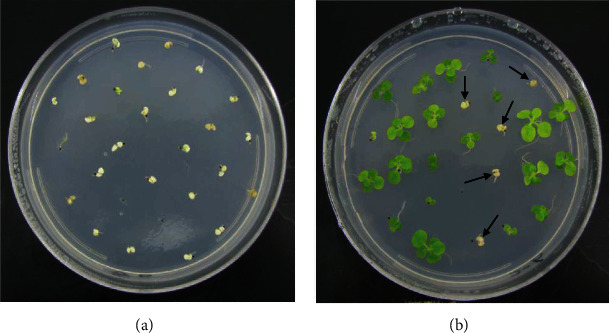
Hygromycin resistance screening of T1 generation of transgenic tobacco seeds with overexpression of *GlaDFR1* and *GlaDFR2*. (a) Chimeric tobacco seeds germinating and dying with 1 or 2 albino leaves developed. (b) Phenotypic separation in transgenic tobacco seeds with the albino seedlings indicated by arrows.

Because the chromosomes have already been replicated or the integrated foreign gene is lost from a chromosome to generate the chimeric plants, which could not maintain the genetic stability in their seeds, in order to verify that the foreign gene integrated in the transgenic tobacco plant maintain its genetic stability, we used the MS medium containing 50 mg/L Hyg to screen the seeds of T1 generation transgenic tobacco plants with overexpression of *GlaDFR1* and *GlaDFR2*. Results showed that although the chimeric seeds could germinate on the selection medium containing Hyg, they only grew 1 or 2 young leaves with the seedlings gradually becoming albino, withered, and died (Figure 15(a)).

The seeds of T1 generation of transgenic tobacco plants showed phenotypic separation (Figure 15(b)). Some seeds grown on MS medium containing 50 mg/L of Hyg germinated with the seedlings showed normal growth with tender leaves and the root system highly developed, while some other seedlings became albino and gradually died. Various ratios of Hyg-resistant and Hyg-sensitive plants were observed in transgenic tobacco plants with overexpression of *GlaDFR1* and *GlaDFR2* (Table 2).

**Figure 16:**
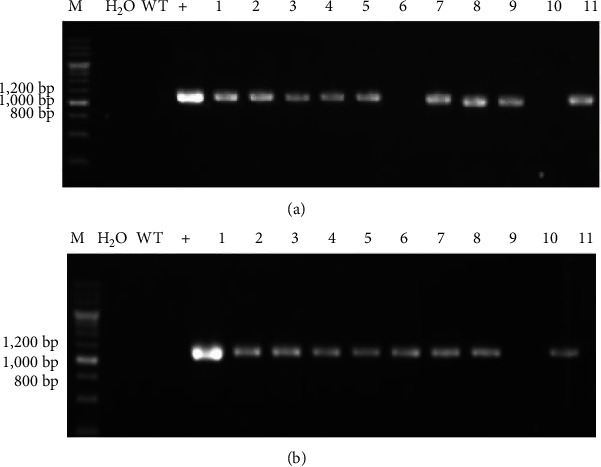
PCR verification of T1 generation of transgenic tobacco seeds with overexpression of (a) *GlaDFR1* and (b) *GlaDFR2*. Lane M represents the 200 bp DNA marker. WT: wild type. Lane “H_2_O” represents the negative control based on ddH_2_O. Lane “+” represents the vector pCAMBIA1302 containing the target genes (i.e., *GlaDFR1* and *GlaDFR2*). Lanes 1–11 represent some of the tobacco plants containing the target gene (~1.1 kb) with blank lanes representing the nontransgenic tobacco plants.

The PCR verification was performed on the T1 generation of the transgenic tobacco plants with overexpression of *GlaDFR1* and *GlaDFR2* using the total DNA extracted from the leaves of WT and T1 generation of transgenic tobacco plants as the templates (Figure 16). Results showed that the T1 generation-resistant tobacco plants were transformed with pCAMBIA1302-GlaDFR1 and pCAMBIA1302-GlaDFR2 vectors, preliminarily indicating that *GlaDFR1* and *GlaDFR2* genes were integrated into the tobacco genome.

**Figure 17:**
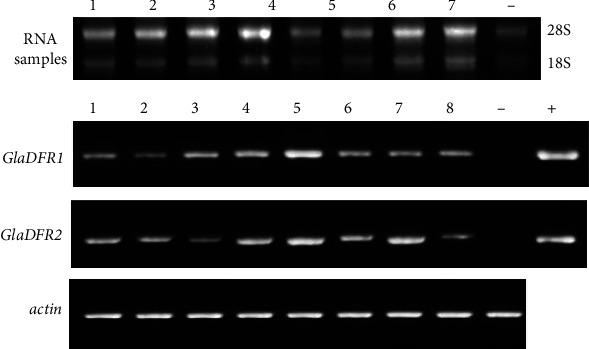
The overexpression of *GlaDFR1* and *GlaDFR2* in transgenic tobacco plants based on RT-PCR with *actin* (EU938079) as the reference gene. Lane “-” represents the wild-type plant. Lane “+” represents the positive control based on vector pCAMBIA1302 containing the target genes (i.e., *GlaDFR1* and *GlaDFR2*). Lanes 1–8 represent 8 transgenic tobacco plants.

In order to further investigate the expression of the target genes, the semiquantitative RT-PCR was performed on tobacco plants with overexpression of *GlaDFR1* and *GlaDFR2* verified as positive by PCR. The quality of the extracted RNA determined the integrity of the cDNA obtained by reverse transcription. The total RNA extracted from WT and transgenic tobacco plants was examined by agar gel electrophoresis (Figure 17). Results showed that both bands of 28S and 18S were clearly recovered with the brightness and width of 28S band as 2 times as those of the 18S band, indicating that the RNA concentration was sufficiently high without any degradation. The concentration of the extracted RNA was determined, ranging from 700 ng/*μ*L to 1600 ng/*μ*L. These results indicated that the extracted RNA was appropriate for the RT-PCR analysis. The results showed that in comparison with WT tobacco, the transgenic tobacco plants with overexpression of *GlaDFR1* and *GlaDFR2* showed the expressions of both target genes at the transcriptional level.

#### 3.5.2. Phenotypic Observation of Petals in T1 Generation of Transgenic Tobacco Plants with Overexpression of *GlaDFR1* and *GlaDFR2*

**Figure 18:**
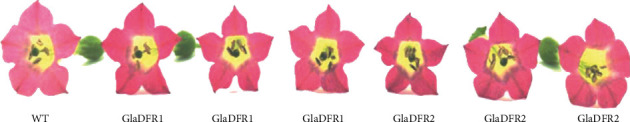
Comparison of the phenotypic observation of petals in wild type (WT) and T1 generation of transgenic tobacco plants with overexpression of *GlaDFR1* and *GlaDFR2.*Bar = 1 cm.

The petals of WT tobacco are generally pink. With the *GlaDFR1* and *GlaDFR2* genes overexpressed in tobacco, it is expected to obtain petals with darker color in the transgenic tobacco plants than that of WT tobacco. Results showed that in comparison to the petal color of WT tobacco plants, the transgenic plants showed darker petals (Figure 18), indicating that the target genes *GlaDFR1* and *GlaDFR2* played a role at the translational level involved in the synthesis of anthocyanidins. As a key enzyme in the biosynthetic pathway of plant flavonoids, the expression level of *DFR* affects the coloring effects of anthocyanidins. Tobacco plants are monoecious and self-pollinating under natural conditions. These results suggested that the overexpression of *GlaDFR1* and *GlaDFR2* in transgenic tobacco plants enhanced the accumulation of pelargonidin in petals. The darker petals were collected, marked, quickly frozen in liquid nitrogen, stored at –80°C, and used for analysis of flavonol metabolites.

#### 3.5.3. Flavonol Metabolites in Petals of T1 Generation of Transgenic Tobacco Plants with Overexpression of *GlaDFR1* and *GlaDFR2*

**Figure 19:**
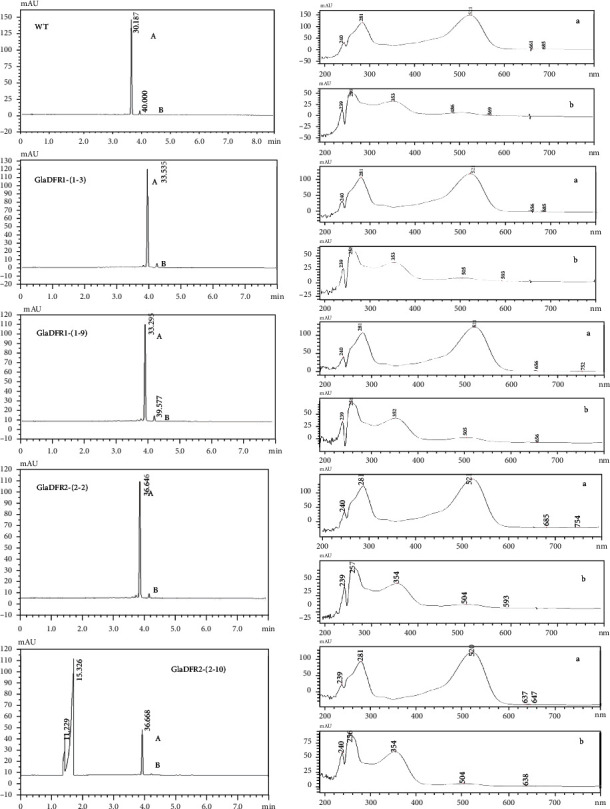
Flavonol metabolites identified by HPLC analysis (520 nm) in petals of wild type (WT) and T1 generation of transgenic tobacco plants with overexpression of *GlaDFR1* (*GlaDFR1*-(1-3) and *GlaDFR1*-(1-9)) and *GlaDFR2* (*GlaDFR2*-(2-2) and *GlaDFR2*-(2-10)). In each sample, the absorption spectra of peaks A and B (left column) are presented in a and b (right column), respectively.

The petals of WT and T1 generation of transgenic tobacco were collected for HPLC analysis of flavonol metabolites (Figure 19). Results showed that the petals of WT tobacco mainly accumulated cyanidin derivatives and a small amount of pelargonidin derivatives. Results of HPLC showed that peaks A and B appeared around 36 min and 40 min, respectively. The results of HPLC-MS showed that peaks A and B represented the same aglycone ion with a mass-to-charge ratio (*m*/*z*) of 286 (Figure S1). Therefore, peaks A and B represented the gentiocyanin derivatives. Based on the previous study with the expression of *Vaccinium macrocarpon* DFR in tobacco [89] and the contents of anthocyanidin in petals, peak A was determined as the monomer of cyanidin 3-arabinoside [89]. The peak area of peak A in transgenic tobacco plants with overexpression of *GlaDFR1* and *GlaDFR2* was reduced in comparison to WT tobacco plants, indicating that the content of gentiocyanin in the petals of transgenic tobacco plants was decreased.

The secondary metabolic pathway of flavonoids in plants has been well studied [18]. As a key enzyme in the biosynthetic pathway of flavonoids, DFR catalyzes 3 types of dihydroflavonols to produce the corresponding leucoanthocyanidins. However, FLS competes with DFR for the dihydroflavonols as substrates to catalyze the formation of flavonols. Our results showed that the WT tobacco accumulated a large amount of quercetin and kaempferol as well as cyanidin and a small amount of pelargonidin in petals. However, no accumulation of myricetin and delphinidin was observed, indicating that WT tobacco petals contained precursor substance for the synthesis of cyanidin and pelargonidin. Therefore, the absence of pelargonidin in tobacco petals was not due to the lack of synthetic precursors but the selection specificity of the endogenous DFR substrates in tobacco. Therefore, tobacco is an ideal experimental material for the functional analysis of *DFR* genes. In our study, we established an efficient and stable *Agrobacterium*-mediated genetic transformation system in tobacco to investigate the function of *DFR* genes based on the overexpression of *GlaDFR1* and *GlaDFR2* genes in the stable genetically transformed tobacco plants.

The compositions of the flavonol metabolites presented in Figure 19 were further analyzed by HPLC-MS (Figure S1; Table 3). The results showed that the flavonols in tobacco were derivatives of quercetin and kaempferol. It was noted that additional studies were needed to determine the two substances revealed at the retention of 22.2 min and 23.2 min, respectively.

The HPLC analysis was performed to examine the contents of anthocyanidins in the petals of transgenic tobacco plants with overexpression of *GlaDFR1* and *GlaDFR2* genes. The results showed that the content of gentiocyanin derivatives in the transgenic tobacco plants was decreased. Results of HPLC-MS analysis showed that the four types of flavonol metabolites were kaempferol and quercetin derivatives, while the content of flavonols was not significantly different from that of the WT, indicating that overexpression of *GlaDFR1* and *GlaDFR2* genes did not affect the changes of flavonols. These results suggested that *GlaDFR1* and *GlaDFR2* genes play important roles in the biosynthesis of anthocyanidins in var. *aurantiaca*. Specifically, GlaDFR1 and GlaDFR2 may be strong competitors for DHK as substrates to produce pelargonidin, thereby inhibiting the catalysis of DHQ to produce cyanidin, ultimately darkening the petals of transgenic tobacco plants. In order to determine the DHK substrate specificity of DFR, our future studies will be focusing on the establishment of prokaryotic expression vectors for the *GlaDFR1* and *GlaDFR2* genes to prepare and purify soluble proteins to investigate the *in vitro* enzymatic activities, providing theoretical evidence for further investigations on the genetic improvement in flower color based on these genes.

## 4. Conclusions

In our study, we amplified both *GlaDFR1* and *GlaDFR2* genes of *G. lutea* L. var. *aurantiaca* from pGEM-GlaDFR1 and pGEM-GlaDFR2 plasmids by PCR technology. Using the binary vector pCAMBIA1302, we constructed the overexpression vectors of *GlaDFR1* and *GlaDFR2* genes (i.e., pCAMBIA1302-GlaDFR1 and pCAMBIA1302-GlaDFR2) regulated by the CaMV 35S promoter and obtained the corresponding engineered *Agrobacterium* strain. Based on the *Agrobacterium*-mediated tobacco leaf disc genetic transformation, the gene overexpression vectors pCAMBIA1302-GlaDFR1 and pCAMBIA1302-GlaDFR2 were transferred into tobacco genome to generate the transgenic tobacco plants with overexpression of *GlaDFR1* and *GlaDFR2*. The results showed that the flower color of T1 generation of transgenic tobacco plants was darker than that of the WT plants. The results of HPLC analysis revealed that the petals of WT tobacco mainly accumulated cyanidin derivatives and a small amount of pelargonidin derivatives, while the contents of cyanidin in the petals of transgenic tobacco plants were decreased. Our study clearly demonstrated that both GlaDFR1 and GlaDFR2 were revealed to show the functions of DFR, providing strong theoretical and experimental evidence to support further studies on the genetic improvement of flower color based on *GlaDFR1* and *GlaDFR2*.

## Figures and Tables

**Table 1:** Characteristics of the primary structure of GlaDFR1 and GlaDFR2 predicted by Protparam.

Characteristics	GlaDFR1	GlaDFR2
Number of amino acids	374	374
Molecular formulae	C_1865_H_2917_N_481_O_571_S_16_	C_1863_H_2915_N_479_O_573_S_16_
Molecular weight (Da)	41,726.52	41,704.46
Isoelectric point	5.22	5.23
Number of negatively charged residues (Asp+Glu)	54	55
Number of positively charged residues (Arg+Lys)	38	39
Instability coefficient	29.24	25.70
Total average hydrophilicity	–0.287	–0.310
Fat factor	84.47	82.91

**Table 2:** Seed segregation in T1 generation of transgenic tobacco plants with overexpression of *GlaDFR1* and *GlaDFR2* selected based on hygromycin resistance screening.

Transgenic tobacco line	Total number of plant	Number of Hyg-resistant plant	Number of Hyg-sensitive plant	Ratio of Hyg-resistant and Hyg-sensitive plants
*GlaDFR1*				
1P1T-2	32	28	4	7 : 1
1P1T-3	32	12	20	0.6 : 1
1P1T-5	32	22	10	2.2 : 1
1P1T-6	32	24	8	3 : 1
1P1T-7	32	25	7	3.6 : 1
1P1T-14	32	24	8	3 : 1
1P1T-16	32	24	8	3 : 1
1P1T-17	32	24	8	3 : 1
*GlaDFR2*				
1P2T-2	32	0	32	0 : 32
1P2T-4	32	0	32	0 : 32
1P2T-5	32	26	6	4.3 : 1
1P2T-6	32	23	9	2.5 : 1
1P2T-7	32	0	32	0 : 32
1P2T-9	32	28	4	7 : 1
1P2T-10	32	25	7	3.6 : 1
1P2T-12	32	25	7	3.6 : 1
1P2T-13	32	25	7	3.6 : 1
1P2T-14	32	24	8	3 : 1

**Table 3:** Contents of flavonol metabolites determined by HPLC-MS analysis (360 nm) in transgenic tobacco plants with overexpression of *GlaDFR1* and *GlaDFR2*.

Peak	Retention (min)	*λ*_max_ (nm)	ESI-MS (*m*/*z*)	Flavonol
1	22.2	249, 323	301.1	Unknown
2	23.2	249, 326	301.1	Unknown
3	31.2	256, 354	302.1[Qr+H]^+^, 465.3[Qr+H+162]^+^, 627.3[Qr+H+162+162]	Quercetin 3-O-glucoside-5-O-glucoside
4	35.5	265, 347	286.9[Km+H]^+^, 449.1[Km+H+162]^+^	Kaempferol 3-O-glucoside
5	48.1	257, 356	303.0[Qr+H]^+^, 465.1[M-100]^+^, 565.5[M+H] ^+^	Quercetin 3-O-succinyl-glucoside
6	55.2	266, 348	287.0[Km+H]^+^, 449.1[Km+H+162]^+^	Kaempferol 5-O-glucoside

## Data Availability

Data are available from the corresponding authors upon request.

## Supplementary Materials

Supplementary Materials Figure S1: identification of the flavonol metabolites based on HPLC-MS analysis (360 nm) in wild-type (WT) and transgenic tobacco plants with overexpression of *GlaDFR1* and *GlaDFR2*.
