# Supplementary material for: Functional Analysis of Genes GlaDFR1 and GlaDFR2 Encoding Dihydroflavonol 4-Reductase (DFR) in Gentiana lutea L. Var. Aurantiaca (M. Laínz) M. Laínz
Source: Biomed Res Int. 2022 Jan 10;2022:1382604. doi: 10.1155/2022/1382604 (PMC8763498; doi:10.1155/2022/1382604)
Supplement: Supplementary Materials — Figure S1: identification of the flavonol metabolites based on HPLC-MS analysis (360 nm) in wild-type (WT) and transgenic tobacco plants with overexpression of GlaDFR1 and GlaDFR2. [file 1382604.f1.pdf]

Figure S1. Identification of the flavonol metabolites based on HPLC-MS analysis (360 nm) in wild type (WT) and transgenic tobacco plants with overexpression of *GlaDFR1* and *GlaDFR2*.

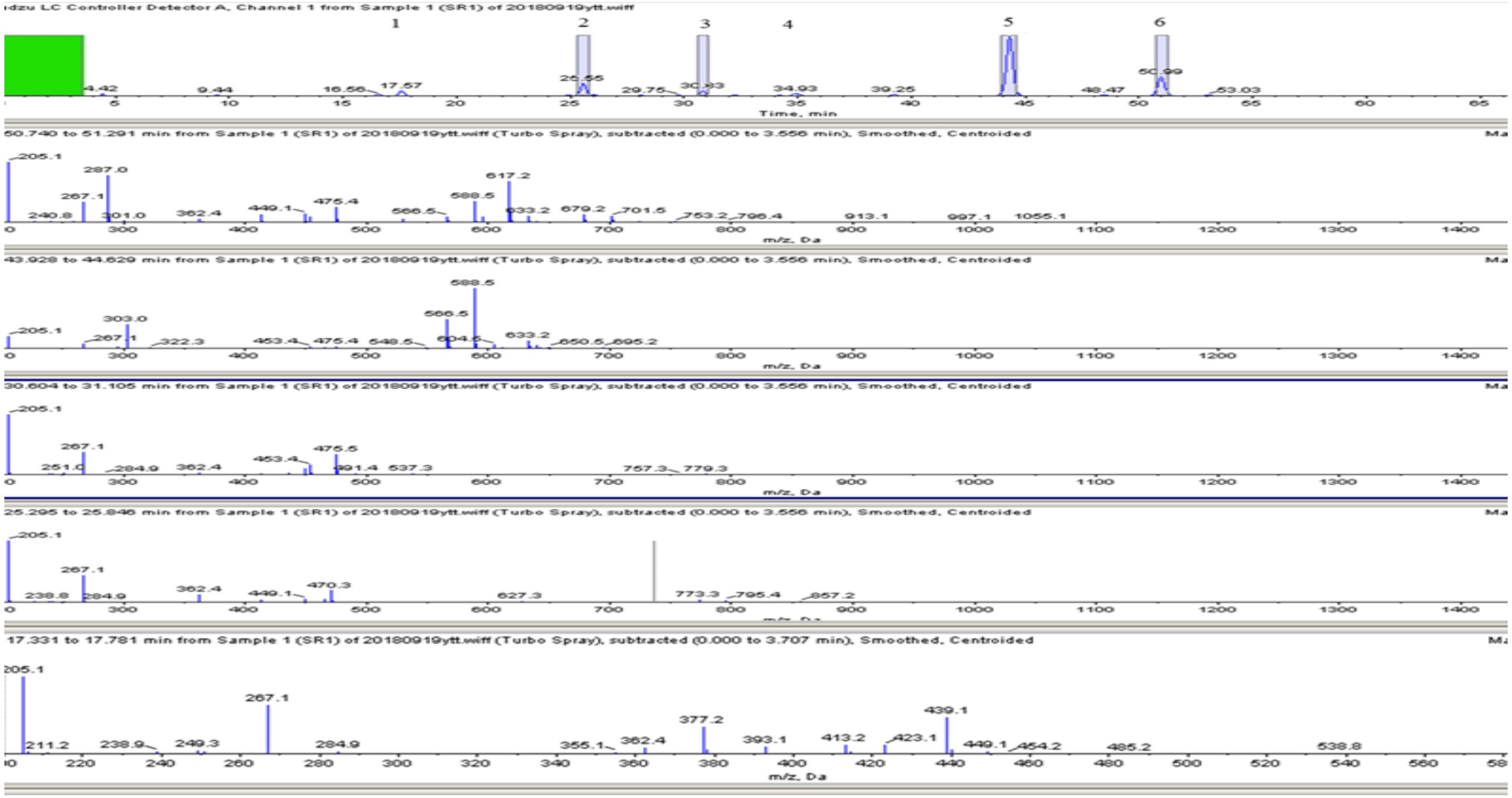

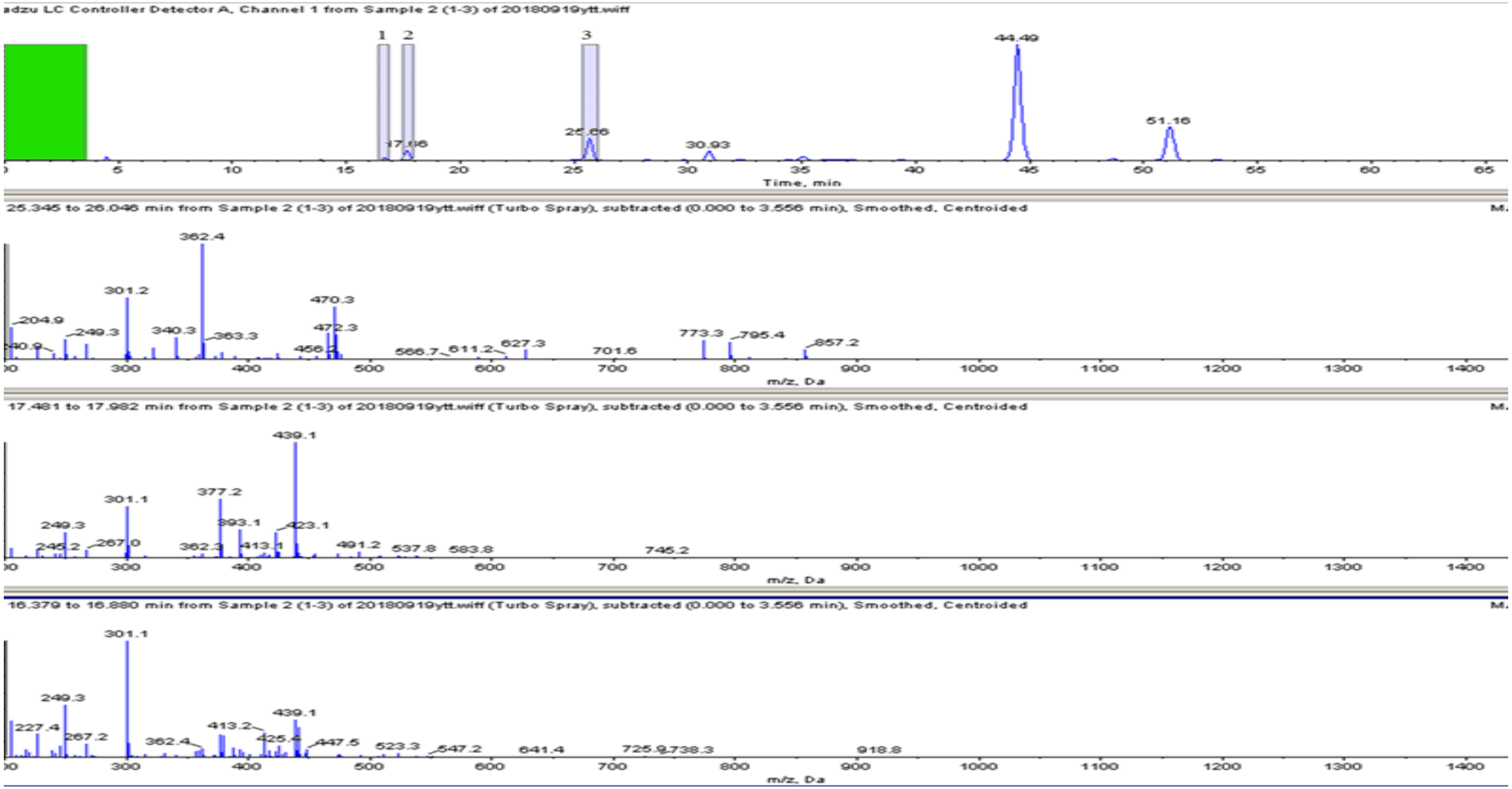

adzu LC Controller Detector A, Channel 1 from Sample 2 (1-3) of 20180919ytt.wiff
1
2
3
44.49
51.16
25.66
30.93
Time, min
25.345 to 26.046 min from Sample 2 (1-3) of 20180919ytt.wiff (Turbo Spray), subtracted (0.000 to 3.556 min), Smoothed, Centroided
362.4
301.2
470.3
204.9
249.3
340.3
363.3
472.3
773.3
795.4
857.2
627.3
611.2
701.6
m/z, Da
17.481 to 17.982 min from Sample 2 (1-3) of 20180919ytt.wiff (Turbo Spray), subtracted (0.000 to 3.556 min), Smoothed, Centroided
439.1
377.2
301.1
249.3
393.1
423.1
491.2
537.8
583.8
745.2
m/z, Da
16.379 to 16.880 min from Sample 2 (1-3) of 20180919ytt.wiff (Turbo Spray), subtracted (0.000 to 3.556 min), Smoothed, Centroided
301.1
249.3
227.4
267.2
362.4
413.2
439.1
447.5
523.3
547.2
641.4
738.3
918.8
m/z, Da

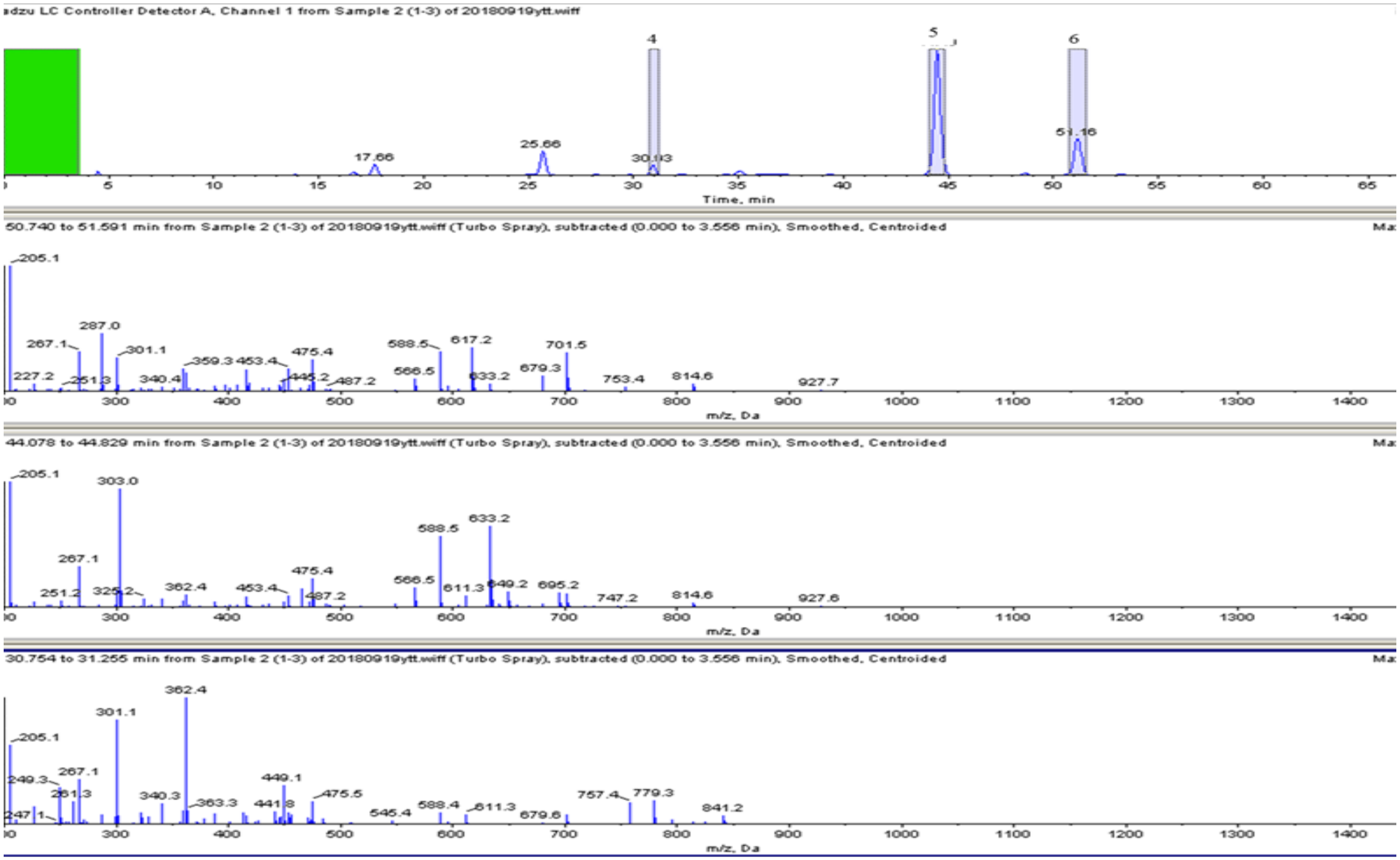

adzu LC Controller Detector A, Channel 1 from Sample 2 (1-3) of 20180919ytt.wiff
4
5
6
17.66
25.66
30.93
51.16
Time, min
50.740 to 51.591 min from Sample 2 (1-3) of 20180919ytt.wiff (Turbo Spray), subtracted (0.000 to 3.556 min), Smoothed, Centroided
205.1
287.0
267.1
301.1
617.2
588.5
701.5
475.4
359.3
453.4
227.2
251.3
340.4
445.2
487.2
566.5
633.2
679.3
753.4
814.6
927.7
m/z, Da
44.078 to 44.829 min from Sample 2 (1-3) of 20180919ytt.wiff (Turbo Spray), subtracted (0.000 to 3.556 min), Smoothed, Centroided
205.1
303.0
633.2
588.5
267.1
475.4
362.4
453.4
251.2
325.2
487.2
566.5
611.3
649.2
695.2
747.2
814.6
927.6
m/z, Da
30.754 to 31.255 min from Sample 2 (1-3) of 20180919ytt.wiff (Turbo Spray), subtracted (0.000 to 3.556 min), Smoothed, Centroided
362.4
301.1
205.1
249.3
267.1
261.3
247.1
340.3
363.3
449.1
441.8
475.5
545.4
588.4
611.3
679.6
757.4
779.3
841.2
m/z, Da

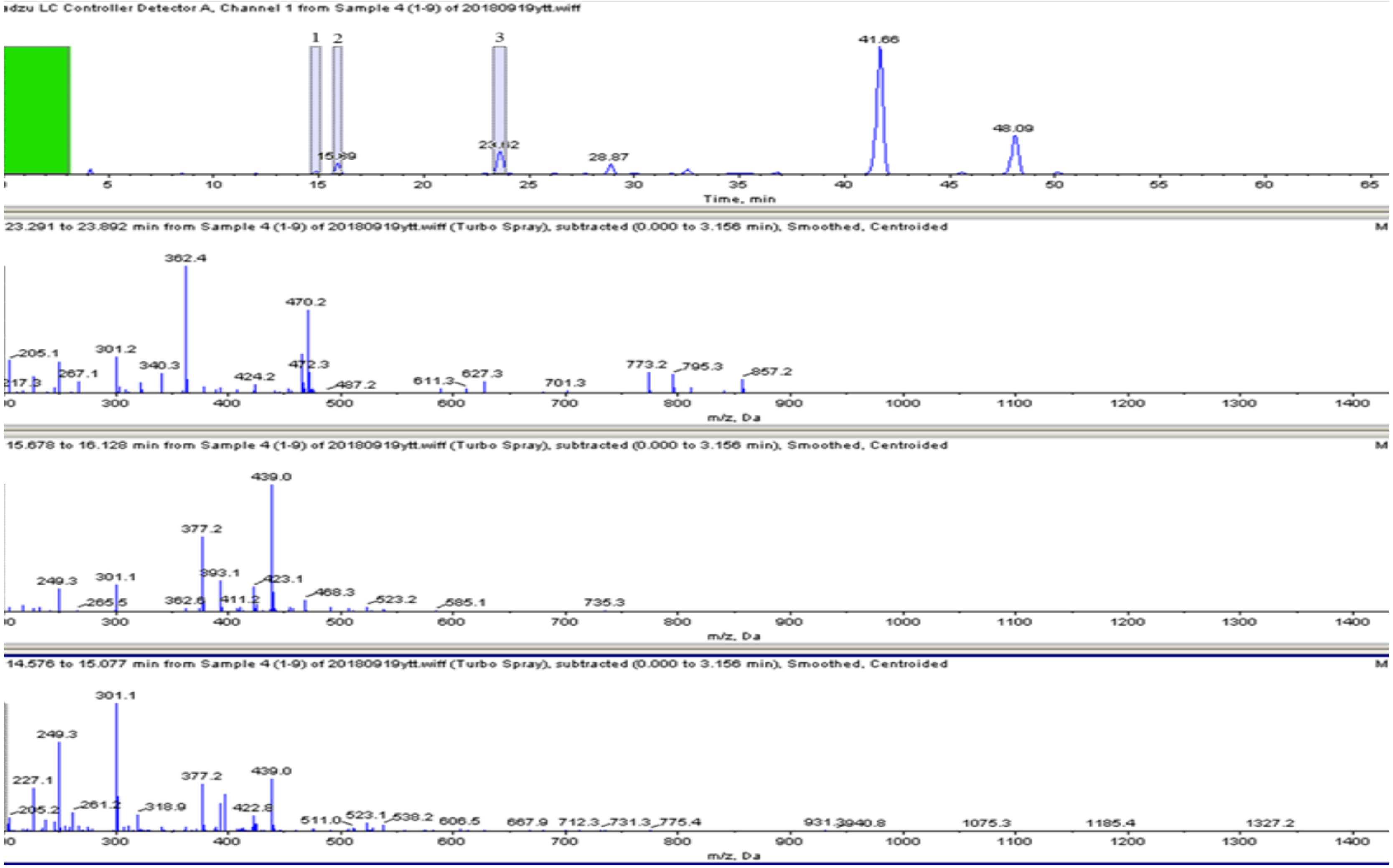
adzu LC Controller Detector A, Channel 1 from Sample 4 (1-9) of 20180919ytt.wiff
1
2
3
41.66
48.09
15.89
23.82
28.87
Time, min
23.291 to 23.892 min from Sample 4 (1-9) of 20180919ytt.wiff (Turbo Spray), subtracted (0.000 to 3.156 min), Smoothed, Centroided
362.4
470.2
205.1
301.2
340.3
267.1
424.2
472.3
487.2
611.3
627.3
701.3
773.2
795.3
857.2
m/z, Da
15.678 to 16.128 min from Sample 4 (1-9) of 20180919ytt.wiff (Turbo Spray), subtracted (0.000 to 3.156 min), Smoothed, Centroided
439.0
377.2
249.3
301.1
393.1
423.1
468.3
523.2
585.1
735.3
m/z, Da
14.576 to 15.077 min from Sample 4 (1-9) of 20180919ytt.wiff (Turbo Spray), subtracted (0.000 to 3.156 min), Smoothed, Centroided
301.1
249.3
227.1
205.2
261.2
318.9
377.2
439.0
422.8
511.0
523.1
538.2
606.5
667.9
712.3
731.3
775.4
1075.3
1185.4
1327.2
m/z, Da

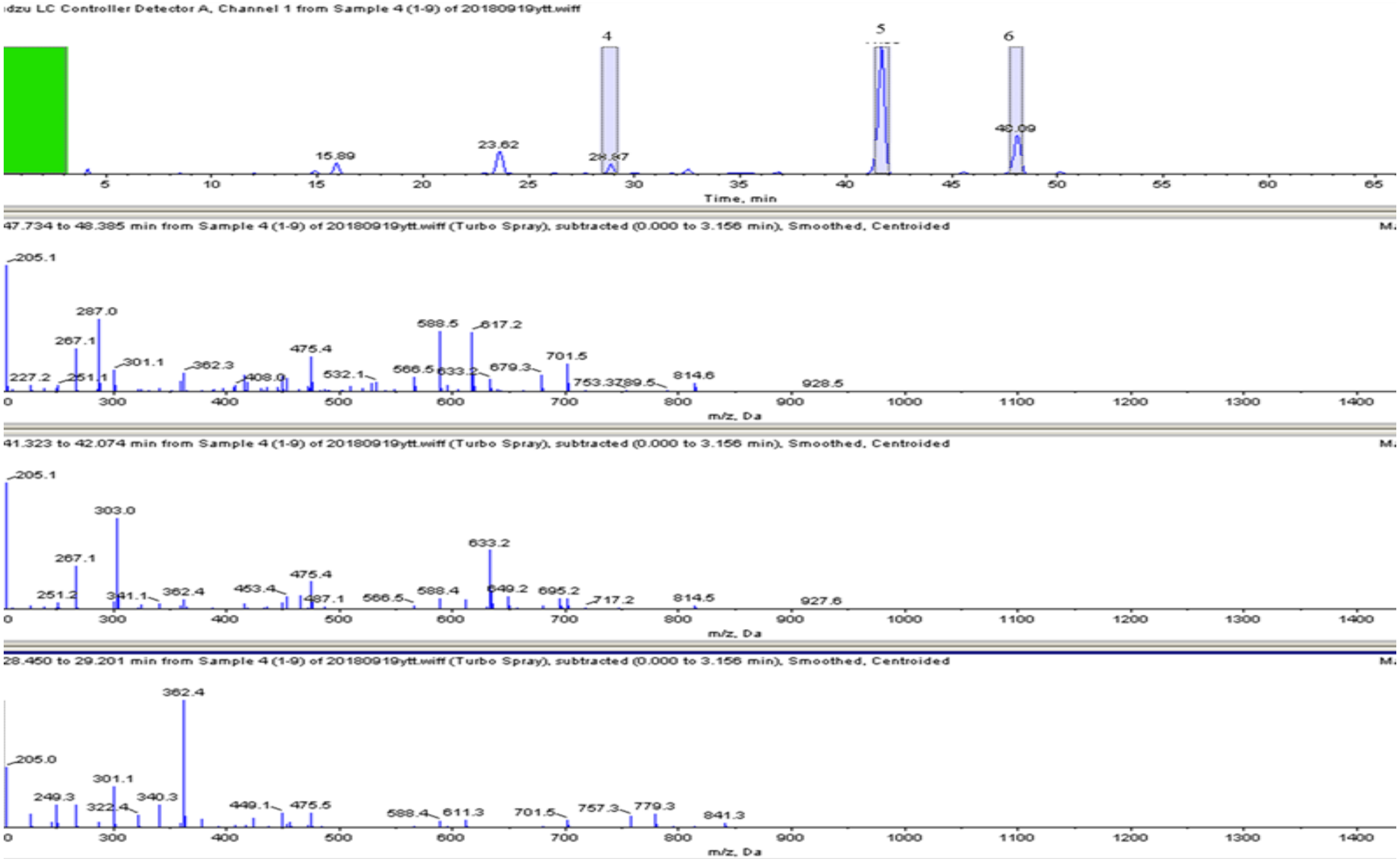
dzu LC Controller Detector A, Channel 1 from Sample 4 (1-9) of 20180919ytt.wiff
4
5
6
15.89
23.62
48.09
Time, min
47.734 to 48.385 min from Sample 4 (1-9) of 20180919ytt.wiff (Turbo Spray), subtracted (0.000 to 3.156 min), Smoothed, Centroided
205.1
287.0
267.1
301.1
362.3
475.4
532.1
566.5
588.5
617.2
679.3
701.5
814.6
928.5
m/z, Da
41.323 to 42.074 min from Sample 4 (1-9) of 20180919ytt.wiff (Turbo Spray), subtracted (0.000 to 3.156 min), Smoothed, Centroided
205.1
303.0
267.1
251.2
362.4
453.4
475.4
487.1
566.5
588.4
633.2
649.2
695.2
717.2
814.5
927.6
m/z, Da
28.450 to 29.201 min from Sample 4 (1-9) of 20180919ytt.wiff (Turbo Spray), subtracted (0.000 to 3.156 min), Smoothed, Centroided
362.4
205.0
301.1
249.3
322.4
340.3
449.1
475.5
588.4
611.3
701.5
757.3
779.3
841.3
m/z, Da

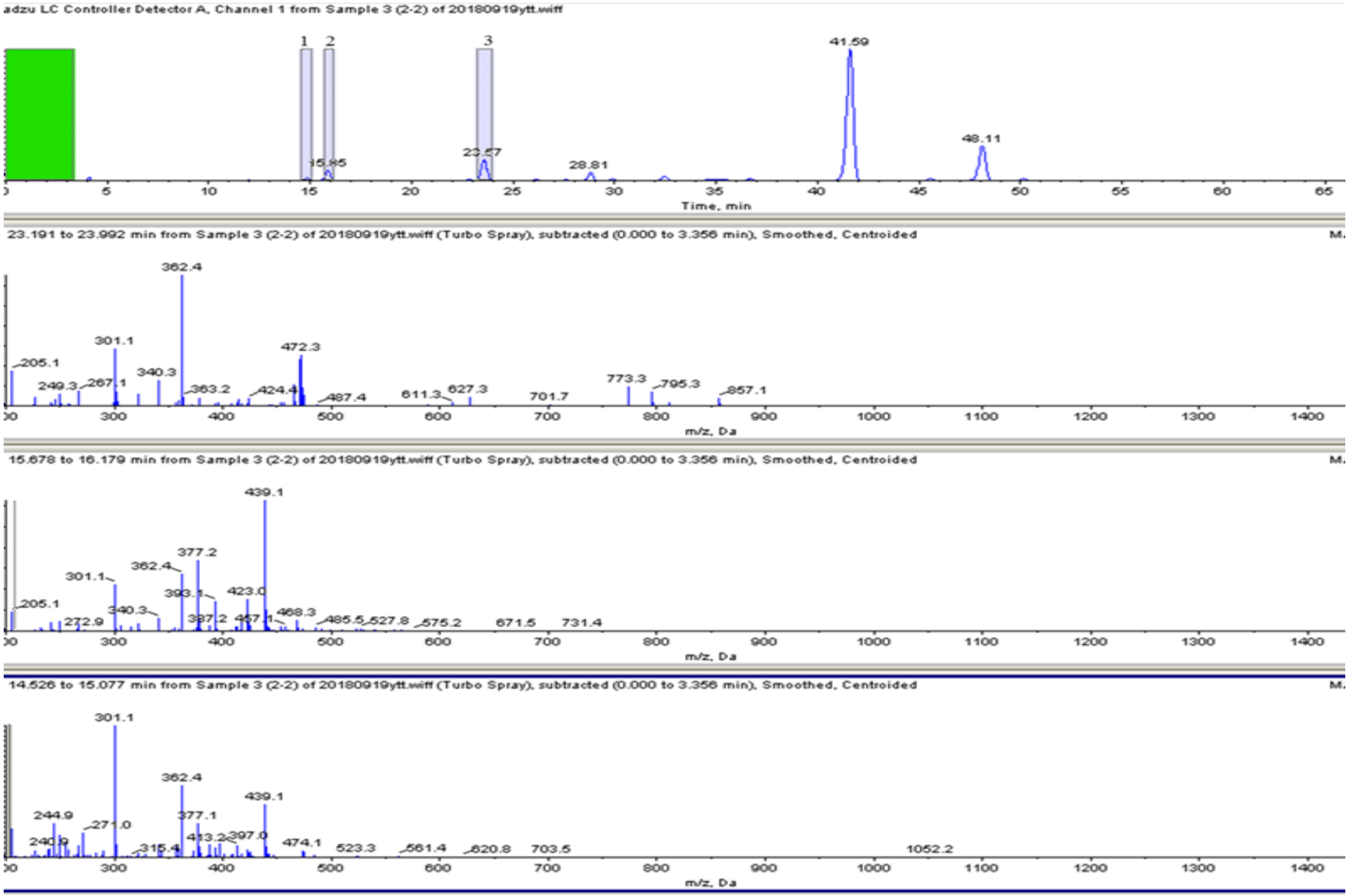
adzu LC Controller Detector A, Channel 1 from Sample 3 (2-2) of 20180919ytt.wiff
Time, min
23.191 to 23.992 min from Sample 3 (2-2) of 20180919ytt.wiff (Turbo Spray), subtracted (0.000 to 3.356 min), Smoothed, Centroided
m/z, Da
15.678 to 16.179 min from Sample 3 (2-2) of 20180919ytt.wiff (Turbo Spray), subtracted (0.000 to 3.356 min), Smoothed, Centroided
m/z, Da
14.526 to 15.077 min from Sample 3 (2-2) of 20180919ytt.wiff (Turbo Spray), subtracted (0.000 to 3.356 min), Smoothed, Centroided
m/z, Da

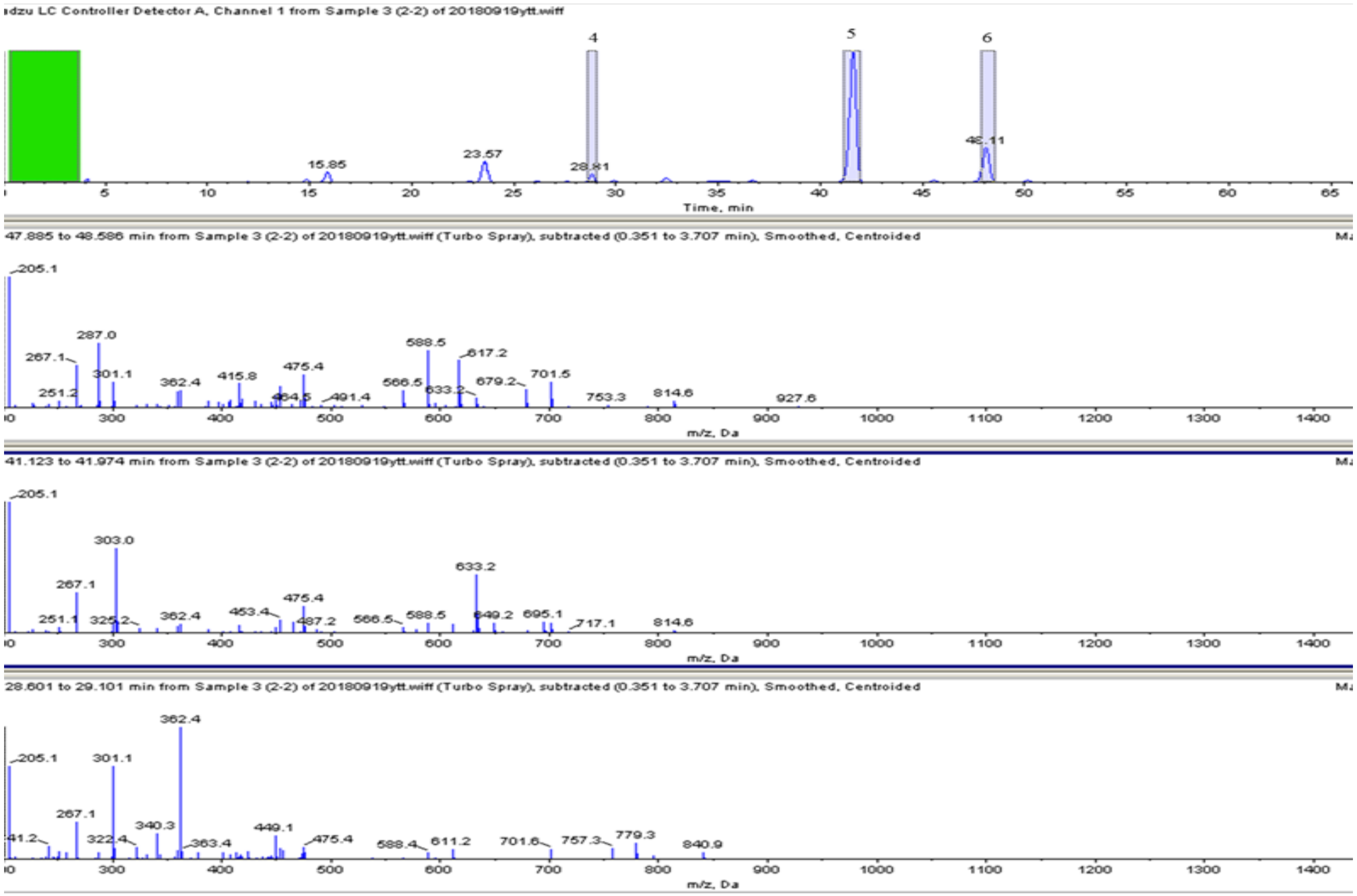

adzu LC Controller Detector A, Channel 1 from Sample 3 (2-2) of 20180919ytt.wiff
4
5
6
15.85
23.57
28.81
48.11
Time, min
47.885 to 48.586 min from Sample 3 (2-2) of 20180919ytt.wiff (Turbo Spray), subtracted (0.351 to 3.707 min), Smoothed, Centroided
205.1
287.0
267.1
301.1
251.2
362.4
415.8
475.4
464.5
491.4
588.5
617.2
566.5
633.2
679.2
701.5
753.3
814.6
927.6
m/z, Da
41.123 to 41.974 min from Sample 3 (2-2) of 20180919ytt.wiff (Turbo Spray), subtracted (0.351 to 3.707 min), Smoothed, Centroided
205.1
303.0
267.1
251.1
325.2
362.4
453.4
475.4
487.2
566.5
588.5
633.2
649.2
695.1
717.1
814.6
m/z, Da
28.601 to 29.101 min from Sample 3 (2-2) of 20180919ytt.wiff (Turbo Spray), subtracted (0.351 to 3.707 min), Smoothed, Centroided
362.4
205.1
301.1
267.1
340.3
241.2
322.4
363.4
449.1
475.4
588.4
611.2
701.6
757.3
779.3
840.9
m/z, Da

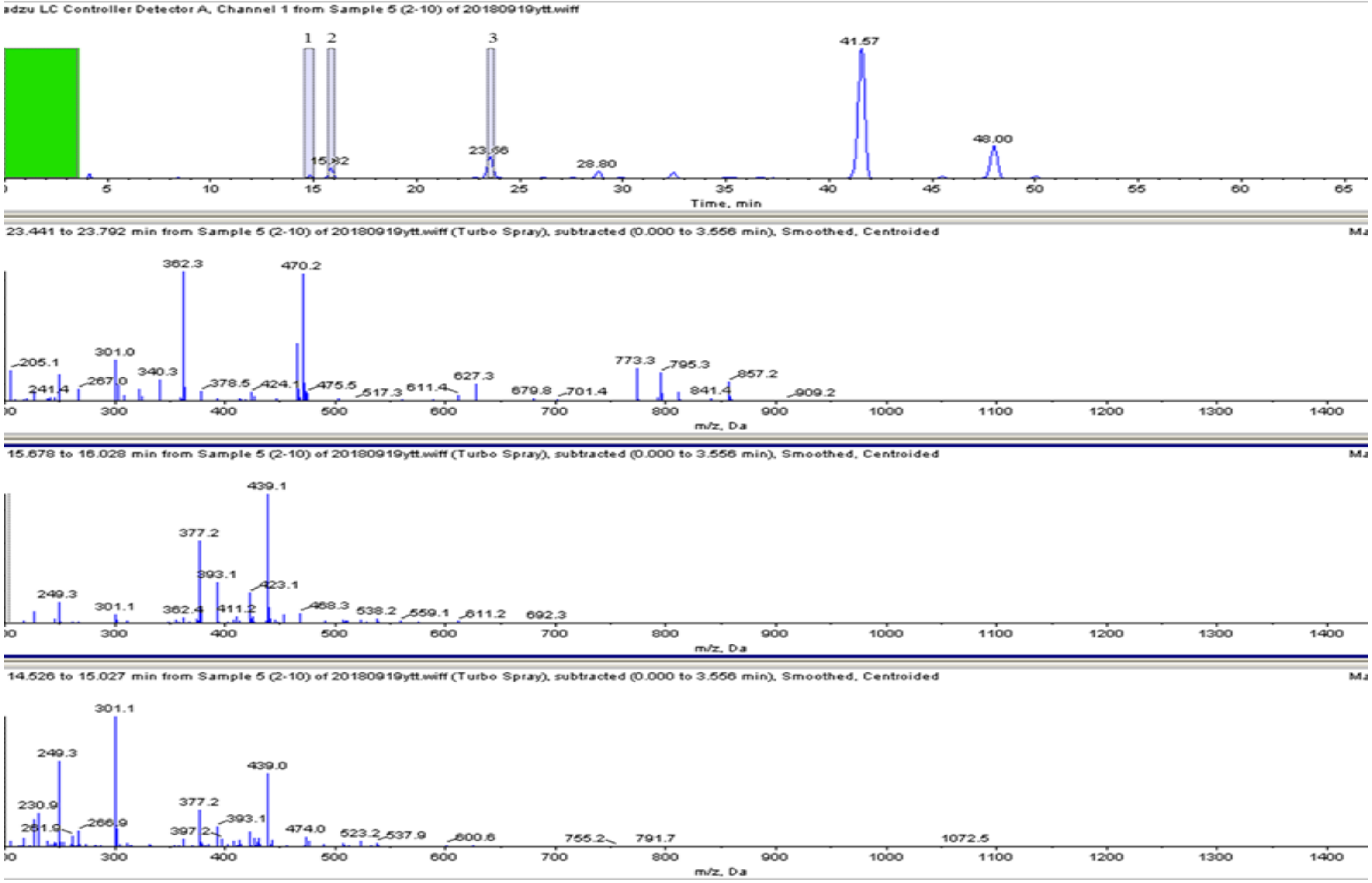
adzu LC Controller Detector A, Channel 1 from Sample 5 (2-10) of 20180919ytt.wiff
1
2
3
41.57
48.00
23.66
15.92
28.80
Time, min
23.441 to 23.792 min from Sample 5 (2-10) of 20180919ytt.wiff (Turbo Spray), subtracted (0.000 to 3.556 min), Smoothed, Centroided
362.3
470.2
205.1
301.0
340.3
241.4
267.0
378.5
424.1
475.5
517.3
611.4
627.3
679.8
701.4
773.3
795.3
841.4
857.2
909.2
m/z, Da
15.678 to 16.028 min from Sample 5 (2-10) of 20180919ytt.wiff (Turbo Spray), subtracted (0.000 to 3.556 min), Smoothed, Centroided
439.1
377.2
393.1
423.1
249.3
301.1
362.4
411.2
468.3
538.2
559.1
611.2
692.3
m/z, Da
14.526 to 15.027 min from Sample 5 (2-10) of 20180919ytt.wiff (Turbo Spray), subtracted (0.000 to 3.556 min), Smoothed, Centroided
301.1
249.3
439.0
230.9
377.2
393.1
266.9
474.0
523.2
537.9
600.6
755.2
791.7
1072.5
m/z, Da

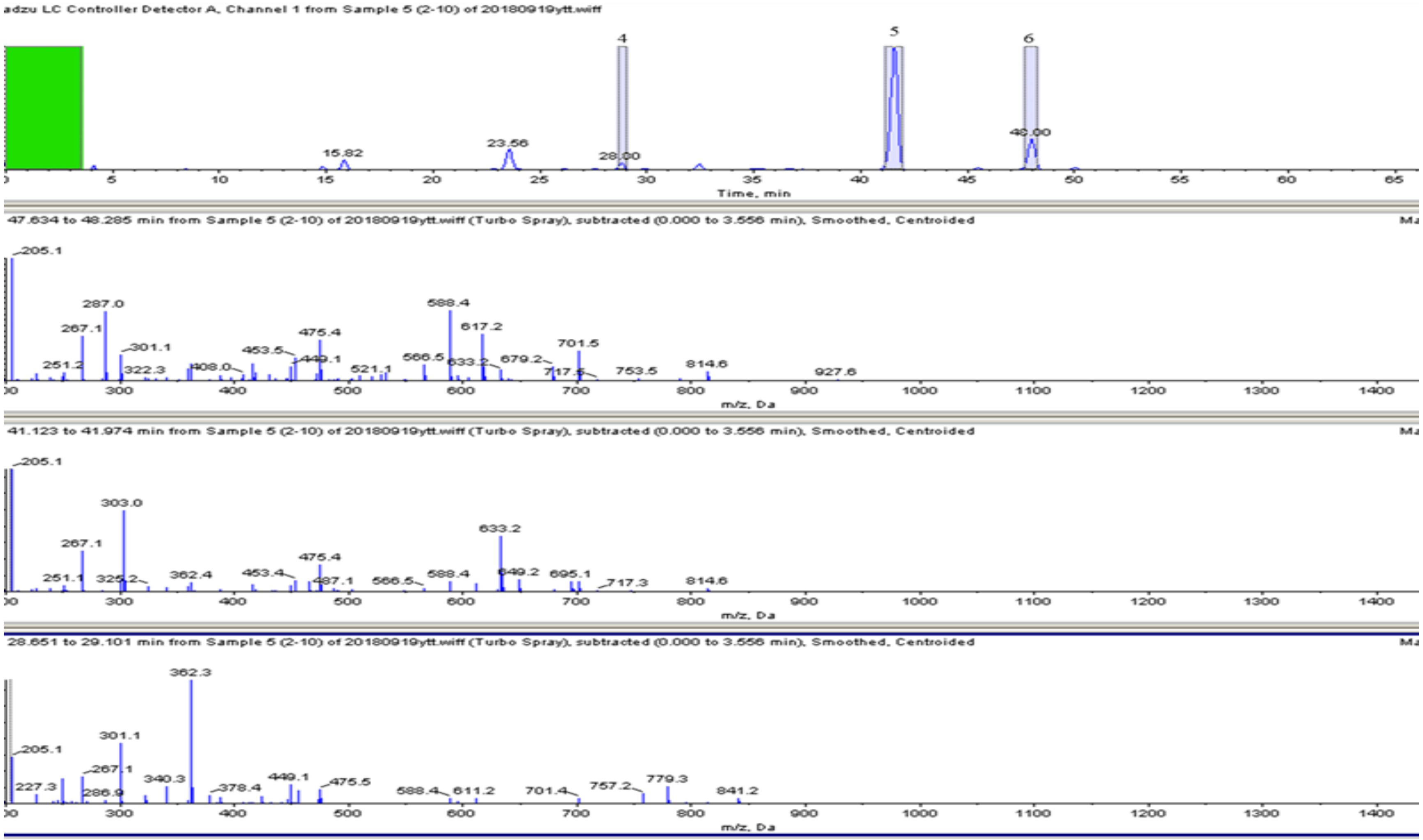
adzu LC Controller Detector A, Channel 1 from Sample 5 (2-10) of 20180919ytt.wiff
4
5
6
15.82
23.56
28.00
48.00
Time, min
47.634 to 48.285 min from Sample 5 (2-10) of 20180919ytt.wiff (Turbo Spray), subtracted (0.000 to 3.556 min), Smoothed, Centroided
41.123 to 41.974 min from Sample 5 (2-10) of 20180919ytt.wiff (Turbo Spray), subtracted (0.000 to 3.556 min), Smoothed, Centroided
28.651 to 29.101 min from Sample 5 (2-10) of 20180919ytt.wiff (Turbo Spray), subtracted (0.000 to 3.556 min), Smoothed, Centroided
m/z, Da
